# Network meta-analysis of the efficacy and safety of monoclonal antibodies and traditional conventional dichotomous agents for chronic obstructive pulmonary disease

**DOI:** 10.3389/fmed.2024.1334442

**Published:** 2024-02-13

**Authors:** Yu Xiong, Jia-qiang Hu, Hui-lin Tang, Zhi-xia Zhao, Li-hong Liu

**Affiliations:** ^1^Institute of Materia Medica, Chinese Academy of Medical Sciences & Peking Union Medical College, Beijing, China; ^2^Department of Pharmacy, China-Japan Friendship Hospital, Beijing, China; ^3^Clinical Trial Research Center, China-Japan Friendship Hospital, Beijing, China; ^4^Personalized Drug Therapy Key Laboratory of Sichuan Province, Department of Pharmacy, Sichuan Provincial People’s Hospital, School of Medicine University of Electronic Science and Technology of China, Chengdu, Sichuan, China; ^5^Department of Pharmaceutical Outcomes and Policy, University of Florida College of Pharmacy, Gainesville, FL, United States

**Keywords:** network meta-analysis, COPD, monoclonal antibodies, Dupilumab, lung function

## Abstract

**Introduction:**

Monoclonal antibodies (mAbs) against cytokines and chemokines or their receptors promise to be a potential therapeutic option to address chronic obstructive pulmonary disease (COPD). We aim to provide a comprehensive literature review of the improvement in FEV1 and safety when comparing mAbs with conventional dichotomous agents.

**Methods:**

We systematically searched 3 electronic databases (PubMed, EMBASE, and CENTRAL) up to August 1, 2023 to collect eligible randomized controlled trials (RCTs). A frequentist network meta-analysis using a random-effects model was deployed to calculate mean differences (MD) for FEV1, relative risk (RR) of treatment-emergent adverse events (TEAEs), and estimate the surface under cumulative rankings (SUCRA). A higher SUCRA indicates a better outcome.

**Results:**

This study included 23 RCTs involving a total of 20,853 patients. Overall, except for Dupilumab, mAbs did not significantly improve FEV1 compared to traditional conventional dichotomous agents. Among all the interventions included, Aclidinium bromide/Formoterol (AB/FF) (SUCRA 97.7%) ranked highest, followed by Umeclidinium/vilanterol (UMEC/VI) (SUCRA 93.5%), and Glycopyrrolate Formoterol Fumarate (GFF) (SUCRA 84.7%). Dupilumab (SUCRA 66.9%) ranked the fourth among all interventions but ranked the first among all the mAbs. Importantly, all mAbs demonstrated a good safety profile compared with placebo.

**Conclusion:**

Considering the improvement in FEV1 and its safety, the development of mAbs for COPD still holds significant clinical potential.

**Systematic review registration:**

PROSPERO, CRD42023452714.

## Introduction

Chronic Obstructive Pulmonary Disease (COPD) is a heterogeneous lung condition characterized by chronic respiratory symptoms (dyspnea, cough, sputum production and/or exacerbations) due to abnormalities of the airways (bronchitis, bronchiolitis) and/or alveoli (emphysema) that cause persistent, often progressive, airflow obstruction (1). According to a large-scale epidemiological study based on the Global Initiative for Chronic Obstructive Lung Disease fixed ratio (GOLD; FEV1/FVC < 0.7) criteria, the global prevalence of COPD is estimated to be 10.3%. Furthermore, with the continued growth of the population and aging in low-income and middle-income countries (LMICs), the prevalence of COPD is expected to rise further (2). COPD poses a significant threat to human health and remains a major cause of death. It is estimated that more than 5.4 million patients will annually succumb to COPD and related diseases by 2060 (3). In addition, COPD places a huge financial burden on patients, their families, and society.

Bronchodilators represent the cornerstone treatments for COPD, and the combination of long-acting muscarinic antagonists (LAMAs) and long-acting β2 agonists (LABAs) has proven to be more effective than monotherapy (4, 5). In line with the 2023 GOLD guidelines, initial treatment for patients in Group B, patients who experience a higher level of symptoms but are at a lower risk of exacerbations, should consist of a combination of LAMA and LABA, and treatment escalation is recommended if symptoms are not adequately controlled on bronchodilator monotherapy (1). In cases of moderate to severe COPD and acute exacerbations, a combination of an inhaled corticosteroid (ICS) with an LABA has demonstrated superior outcomes in improving lung function and health status as well as reducing exacerbations when compared to using either component alone (6, 7).

Inflammatory responses play a pivotal role in COPD, with numerous inflammatory mediators, including lipid mediators, cytokines, chemokines, and peptides, contributing to the complex inflammatory processes observed in this condition. These mediators are responsible for the recruitment and activation of inflammatory cells, as well as the structural changes that occur over time (8). Unfortunately, inflammation in COPD is often resistant to corticosteroid treatment (9). As a result, identifying effective and well-tolerated anti-inflammatory drugs for COPD patients remains a significant challenge (10). Monoclonal antibodies (mAbs) targeting cytokines and chemokines or their receptors show promise as potential therapeutic options for addressing the inflammatory component of COPD (11), given their success in treating chronic inflammatory diseases such as severe asthma, rheumatoid arthritis, and inflammatory bowel disease (9). Despite COPD patients receiving inhaled drug therapy, there remains a risk of lung function decline and exacerbations. Therefore, this study aims to compare the effects of adding mAbs therapy to inhaled drug therapy with dual therapy on pulmonary function in COPD patients, expecting to provide additional evidence for the use of mAbs in patients with COPD.

## Methods

This was a Network meta-analysis (NMA) of the efficacy and safety of mAbs and conventional dual therapy agents in COPD patients. We conducted this NMA following the guidelines provided by the Preferred Reporting Items for Systematic Reviews and Meta-analysis (PRISMA) statement. Our study was registered with PROSPERO (CRD42023452714).

### Literature search strategy

We performed a comprehensive search of three databases including PubMed, EMBASE, and the Cochrane Central Register of Controlled Trials (CENTRAL) from the inception until August 2023 to identify randomized controlled trials (RCTs) that evaluated mAbs or dual therapy for COPD. The search keywords included “Pulmonary Disease, Chronic Obstructive,” “Chronic Obstructive Pulmonary Diseases,” “COPD,” “Formoterol,” “Glycopyrrolate,” “Monoclonal antibody,” “Benralizumab,” “Mepolizumab” and “Dupilumab” etc. as MeSH and free text terms. The search was conducted without any language restrictions Details regarding the search strategies were shown in Supplementary Table S1.

### Study selection

We included RCTs that met the following criteria: (1) Trials including patients with COPD; (2) Receiving dual therapy (budesonide/formoterol (BF), umeclidinium/vilanterol (UMEC/VI), fluticasone furoate/ vilanterol (FF/VI), glycopyrrolate formoterol fumarate (GFF), and aclidinium bromide formoterol (AB/FF)) or mAbs (benralizumab, mepolizumab, reslizumab, canakinumab, ABX-IL8, infliximab, etanercept, itepekimab, astegolimab, lebrikizumab, CNTO-6785, MEDI-8986, AMG-282, tozorakimab, and dupilumab) in combination with conventional therapy; and (3) Trials reporting the change in forced expiratory volume in the first second (FEV1) from baseline among patients at the end of the treatment period were included. Studies meeting the following criteria were excluded: (1) Duplicate publications; (2) Trials with no relevant data and inconsistent outcome measures. Two reviewers independently screened titles and abstracts, and reviewed full texts to decide on studies to be included. Any discrepancies regarding study selection were resolved by consensus or consultation with a third reviewer.

### Data extraction and quality assessment

For every included study, the independent reviewer extracted the following data: (1) Basic information of included studies: study title, first author and number of included patients; (2) Baseline characteristics of study population: age, sex, smoking status, proportion of patients with severe and very severe COPD, mean pre-bronchodilator FEV1, mean post-bronchodilator FEV1, mean pre-bronchodilator FEV1% predicted (Pre-FEV1%), mean post-bronchodilator FEV1% predicted (Post-FEV1%); (3) Intervention measures: type of intervention and duration of treatment; and (4) The outcomes of interest: change in FEV1 from baseline among patients at the end of the treatment period and the risk of adverse events (AEs), and the change in FEV1 is in litres (L).

Two reviewers(YX and JQH) independently assessed the risk of bias of included trials using the Version 2 of the Cochrane tool for assessing risk of bias in randomized trial(RoB-2) (12, 13).

### Statistical analyses

Statistical analysis was performed using Stata 17.0 and R 4.2.3 software. A frequentist NMA using a random-effects model was deployed to calculate mean differences (MDs) with 95% confidence intervals (CIs) for FEV1 and calculate relative risk (RR) with 95% CI for the incidence of treatment-emergent adverse events (TEAEs). The efficacy and safety of each drug intervention regimen for each outcome was predicted using the surface under the cumulative ranking curve (SUCRA). The assessment of the inconsistency of the network was unavailable because the network in our analysis was star-shaped and did not have a closed loop. The level of statistical significance was set as *p* < 0.05.

## Results

### Study selection and study characteristics

**Figure 1:**
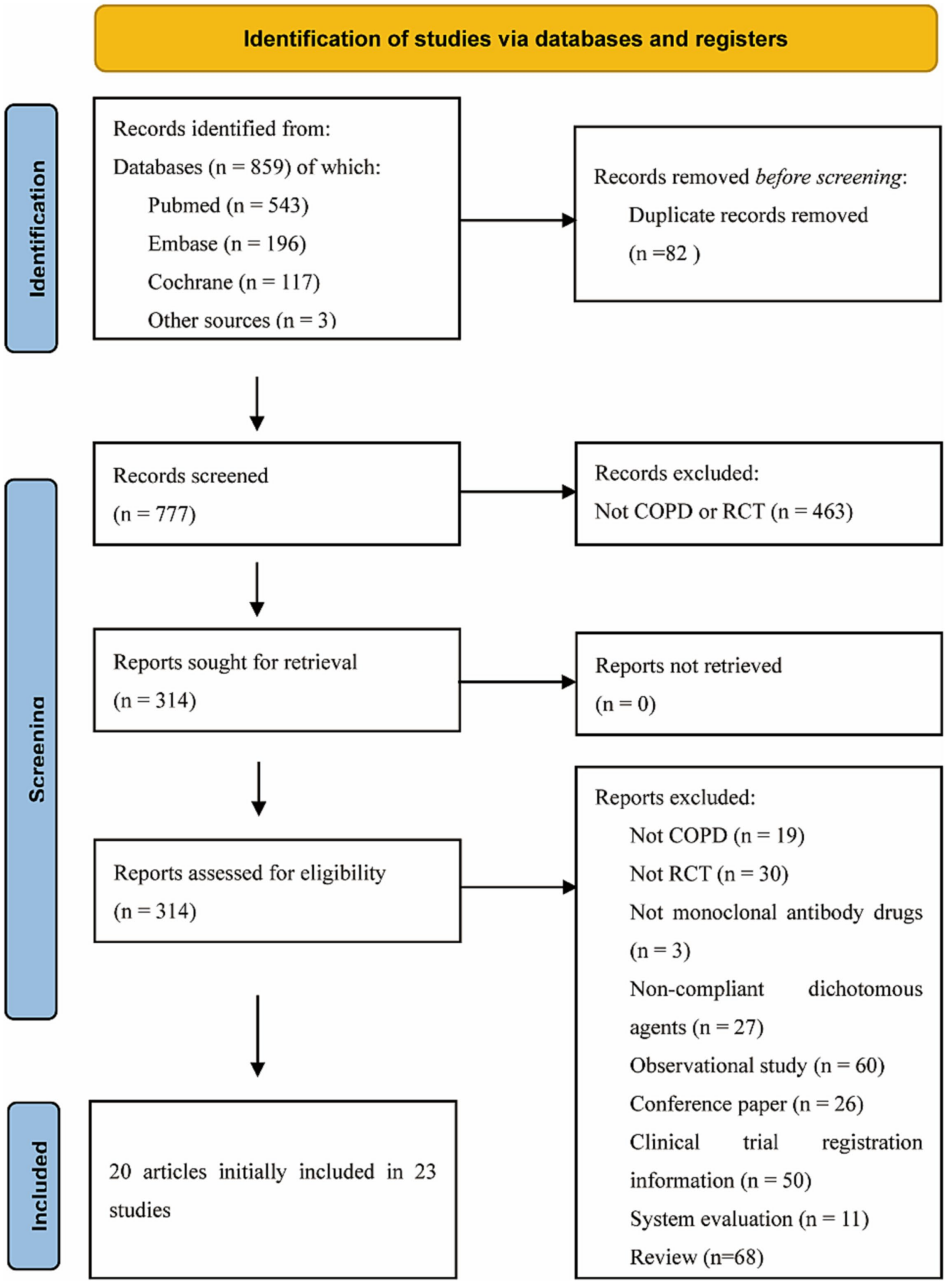
Process for identifying studies eligible for the network meta-analysis.

The process of study selection is presented in the PRISMA study flow diagram (Figure 1). A total of 859 relevant articles were included in the initial search, and 82 duplicate studies and 463 unrelated studies were excluded during the screening of titles and abstracts. After reviewing the full texts of 314 articles, a total of 20 articles including 23 RCTs that met the criteria were included, of which 13 were dual therapy RCTs and 10 were mAbs therapy RCTs. A total of 13 interventions (ABX-IL8, Benralizumab, Mepolizumab, Itepekimab, Astegolimab, Dupilumab, MEDI-8968, CNTO-6785, BF, UMEC/VI, GFF, AB/FF, FF/VI) that were compared with placebo were included, and the characteristics of the studies and patients are shown in Table 1.

**Table 1:** Baseline characteristics of the included studies.

Author, year	NCT number	Treatment	Sample size, *n*	Age, years	Male, %	Current smokers, %	Severe or very severe COPD, %	Mean pre-bronchodilator FEV1, L	Mean post-bronchodilator FEV1, L	Mean Pre-FEV1%	Mean Post-FEV1%	Duration, weeks	Key endpoints
Koopman, 2022 (14)	NCT02424344	AB/FF 400/12 ug BID	126	63.0	62.0	64.0	NR	1.50	NR	NR	NR	4	FEV1, FVC, FRC, AEs, etc.
		Placebo	124	62.0	58.0	62.0	NR	1.60	NR	NR	NR		
Lipworth, 2018 (15)	NCT02343458	GFF18/9.6 ug BID	551	64.7	74.0	45.7	NR	NR	NR	NR	53.96	24	FEV1, TDI focal score, SGRQ total score, AEs, etc.
		Placebo	235	64.0	72.8	48.1	NR	NR	NR	NR	54.40		
Reisner, 2017 (16)	NCT02347072	GFF18/9.6 ug BID	73	61.9	35.6	61.6	27.4	1.41	1.54	52.54	57.69	4	FEV1, IC, AEs, etc.
		Placebo	69	61.7	34.8	62.3	31.9	1.40	1.54	51.70	56.61		
	NCT02347085	GFF18/9.6 ug BID	35	61.3	57.1	57.1	40.0	1.41	1.53	48.34	52.51		
		Placebo	35	61.3	57.1	57.1	40.0	1.41	1.53	48.34	52.51		
Martinez, 2017 (17)	NCT01854645	GFF18/9.6 ug BID	526	62.6	55.1	53.4	46.0	NR	1.50	NR	51.40	24	FEV1, SGRQ total score, daily rescue albuterol use, AEs, etc.
		Placebo	219	62.5	55.7	57.5	47.0	NR	1.50	NR	50.60		
	NCT01854658	GFF 8/9.6 ug BID	510	62.8	53.3	52.5	47.7	NR	1.50	NR	52.10		
		Placebo	223	64.2	56.1	49.3	47.5	NR	1.50	NR	52.50		
Vestbo, 2016 (18)	NCT01313676	FF/VI 100/25 ug qd	4,121	65.0	76.0	45.0	NR	NR	1.70	NR	59.70	144	FEV1, exacerbations rate, AEs, etc.
		Placebo	4,111	65.0	75.0	47.0	NR	NR	1.70	NR	59.70		
Zheng, 2015 (19)	NCT01636713	UMEC/VI 125/25 ug qd	193	63.7	94.0	25.0	NR	NR	1.20	NR	NR	24	FEV1, TDI focal score, rescue-albuterol use, time to first COPD exacerbation, AEs, etc.
		UMEC/VI 62.5/25 ug qd	194	64.0	94.0	29.0	NR	NR	1.13	NR	NR		
		Placebo	193	64.3	92.0	34.0	NR	NR	1.17	NR	NR		
Maltais, 2014 (20)	NCT01323660 NCT01328444	UMEC/VI 125/25 ug qd UMEC/VI 62.5/25 ug qd Placebo	655	62.0	55.4	62.0	NR	NR	NR	NR	NR	12	EET, FEV1, AEs, exacerbations rate, etc.
Celli, 2014 (21)	NCT01313637	UMEC/VI 125/25 ug qd	403	63.4	66.0	50.0	NR	NR	NR	NR	47.70	24	FEV1, TDI score, SGRQ total score, AEs, etc.
		Placebo	275	62.2	64.0	52.0	NR	NR	NR	NR	47.60		
Donohue, 2013 (22)	NCT01313650	UMEC/VI 62.5/25 ug qd	413	74.0	74.0	49.0	51.0	NR	NR	NR	47.80	24	FEV1, FVC, TDI focal score, SGRQ score, AEs, etc.
		Placebo	280	62.2	70.0	54.0	59.0	NR	NR	NR	46.70		
Tashkin, 2008 (23)	NCT00206154	BF 320/9 ug bid	277	63.1	67.9	44.4	NR	1.04	NR	NR	39.05	26	FEV1, SGRQ total score, time-to-first COPD exacerbation, AEs, etc.
		BF 160/9 ug bid	281	63.6	64.4	44.8	NR	1.04	NR	NR	39.87		
		Placebo	300	63.2	69.0	39.7	NR	1.08	NR	NR	41.28		
Mahler, 2004 (24)	NR	ABX-IL8 10 mg/mL 3/4w	56	65.0	62.0	NR	39.0	NR	NR	NR	NR	13	TDI total score, SGRQ total score, FEV1, exacerbations rate, AEs, etc.
		Placebo	53	63.0	47.0	NR	40.0	NR	NR	NR	NR		
Brightling, 2014 (25)	NCT01227278	Benralizumab 100 mg Q4W/Q8W	51	62·9	69.0	33.0	52.0	1.30	1.50	NR	NR	56	Exacerbations rate, SGRQ scores, FEV1, AEs, etc.
		placebo	50	64.6	58.0	42.0	38.0	1.40	1.50	NR	NR		
Criner, 2019 (26)	NCT02138916	Benralizumab 30 mg Q8W	382	65.8	70.7	36.6	NR		1.203	39.7	42.4	56	Exacerbations, FEV1, SGRQ total score, AEs.
		Benralizumab 100 mg Q8W	379	65.5	69.1	34.0	NR		1.234	40.5	43.5		
		Placebo	359	65.6	72.4	32.0	NR		1.237	41.1	43.3		
	NCT02155660	Benralizumab 10 mg Q8W	377	65.1	66.8	28.6	NR		1.204	42.7	43.7	56	
		Benralizumab 30 mg Q8W	394	65.9	68.3	27.4	NR		1.152	40.5	42.6		
		Benralizumab 100 mg Q8W	385	64.9	64.8	28.0	NR		1.175	40.9	42.5		
		Placebo	388	65.0	65.2	30.4	NR		1.171	41.2	42.9		
Rabe, 2021 (27)	NCT03546907	Itepekimab 300 mg Q2W	172	63.7	58.0	43.0	NR	1.30	1.40	45.70	49.60	24–52	Exacerbations RATE, FEV1, AEs, etc.
		Placebo	171	64.0	56.0	48.0	NR	1.30	1.40	45,6	49.00		
Pavord, 2021 (28)	NCT02105948 NCT02105961	Mepolizumab 100 mg Q4W	456	65.0	62.0	26.0	62.0	1.16	NR	42.90	NR	52	Exacerbation rate, FEV1, SGRQ total score, CAT scores, AEs, etc.
		Placebo	455	66.0	67.0	33.0	65.0	1.14	NR	40.90	NR		
		Mepolizumab 100 and 300 mg combined	681	65.0	64.0	72.0	62.0	1.16	NR	42.60	NR		
		Placebo	455	66.0	67.0	70.0	65.0	1.14	NR	40.90	NR		
Yousuf, 2022 (29)	NCT03615040	Astegolimab 490 mg Q4W	42	67.6	60.0	24.0	NR	1.10	1.20	NR	48.20	48	Exacerbations rate, FEV1, SGRQ total score, CAT scores, AEs, etc.
		Placebo	39	70.8	67.0	15.0	NR	1.00	1.10	NR	44.90		
Eich, 2017 (30)	NCT01966549	CNTO 6785 6 mg/kg (>100 kg, 600 mg), 0, 2, 4, 8, 12w	93	62.0	65.6	49.5	32.6	1.57	1.70	51.92	56.18	16	Percent-predicted FEV1, FEV1, AEs, etc.
		Placebo	94	62.4	69.1	42.6	45.7	1.52	1.64	50.07	53.92		
Calverley, 2017 (31)	NCT01448850	MEDI-8968 600 mg (loading dose), 300 mg Q4W	160	62.8	68.8	NR	76.9	1.20	1.30	39.70	41.90	52	Exacerbation rate, FEV1, SGRQ total score, AEs, etc.
		Placebo	164	63.0	67.1	NR	73.2	1.10	1.20	38.60	42.10		
Dasgupta, 2017 (32)	NCT01463644	Mepolizumab 750 mg/month	8	NR	NR	NR	NR	1.35	1.53	55.00	58.50	24	Sputum eosinophil percentage, exacerbation rate, FEV1.
		Placebo	10	NR	NR	NR	NR	0.99	1.24	29.50	35.00		
Bhatt, 2023 (33)	NCT03930732	Dupilumab 300 mg Q2W	468	65.0	63.7	28.6	NR	1.28	1.39	NR	NR	52	Percent-predicted FEV1, FEV, SGRQ total score, AEs, etc.
		placebo	471	65.2	68.4	31.4	NR	1.32	1.41	NR	NR		

NR, not reported. FRC, functional residual capacity; FVC, forced vital capacity; IC, inspiratory capacity; EET, exercise endurance time; SGRQ, St George’s Respiratory Questionnaire; CAT, COPD Assessment test; TDI, Transition Dyspnea Index; AEs, adverse events.

### Risks of bias

**Figure 2:**
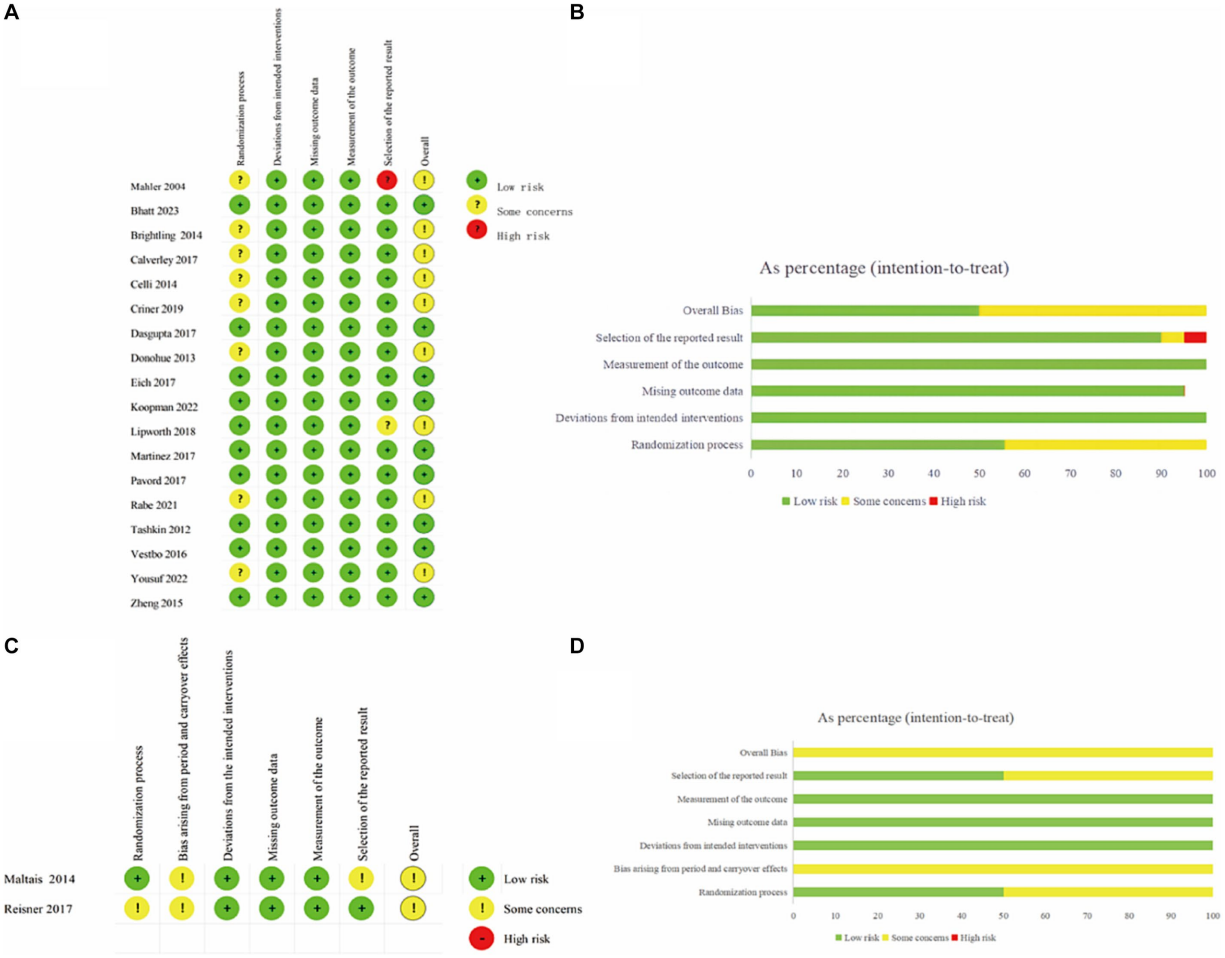
Risk of bias assessment of RCTs using the ROB-2 tool. **(A)** Traffic light plot in individually-randomized and parallel-group trials. **(B)** Weighted summary plot of the overall type of bias encountered in individually-randomized and parallel-group trials. **(C)** Traffic light plot in crossover trials. **(D)** Weighted summary plot of the overall type of bias encountered in crossover trials.

**Figure 3:**
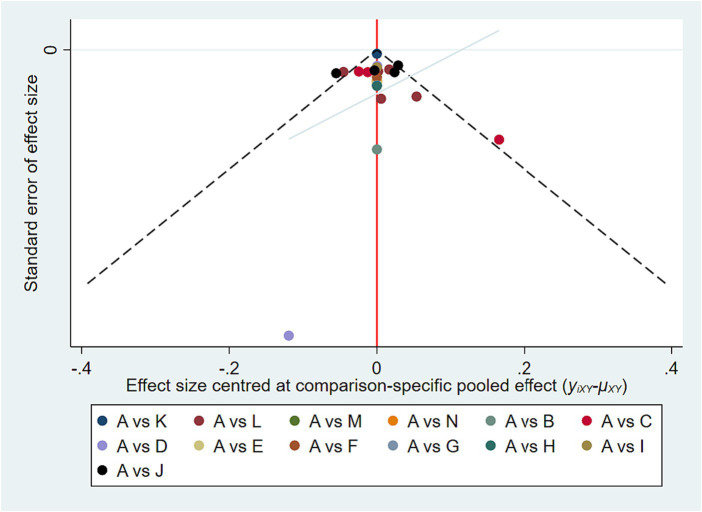
Funnel plot of publication bias. A, Placebo; B, ABX-IL8; C, Benralizumab; D, Mepolizumab; E, Itepekimab; F, Astegolimab; G, MEDI-8968; H, CNTO-6785; I, BF; J, UMEC/VI; K, FF/VI; L, GFF; M, AB/FF; N, Dupilumab.

Eleven trials exhibited a low risk of bias on the randomization process, whereas 9 studies had “some concerns” for this domain. Both cross-over trials appear as “some concerns” in the domain of Bias arising from period and carryover effects. No studies showed a high risk in Deviations from the intended interventions, Missing outcome data and Measurement of the outcome. In terms of Selection of the reported results, one study was high risk and two studies had “some concerns.” In the overall risk-of-bias judgment, we classified nine studies as “low” and eleven as “some concerns.” The quality assessment of the articles included in the meta-analysis was summarized in Figure 2. Funnel plots suggested there was no publication bias among these studies (Figure 3).

### Network meta-analysis

**Figure 4:**
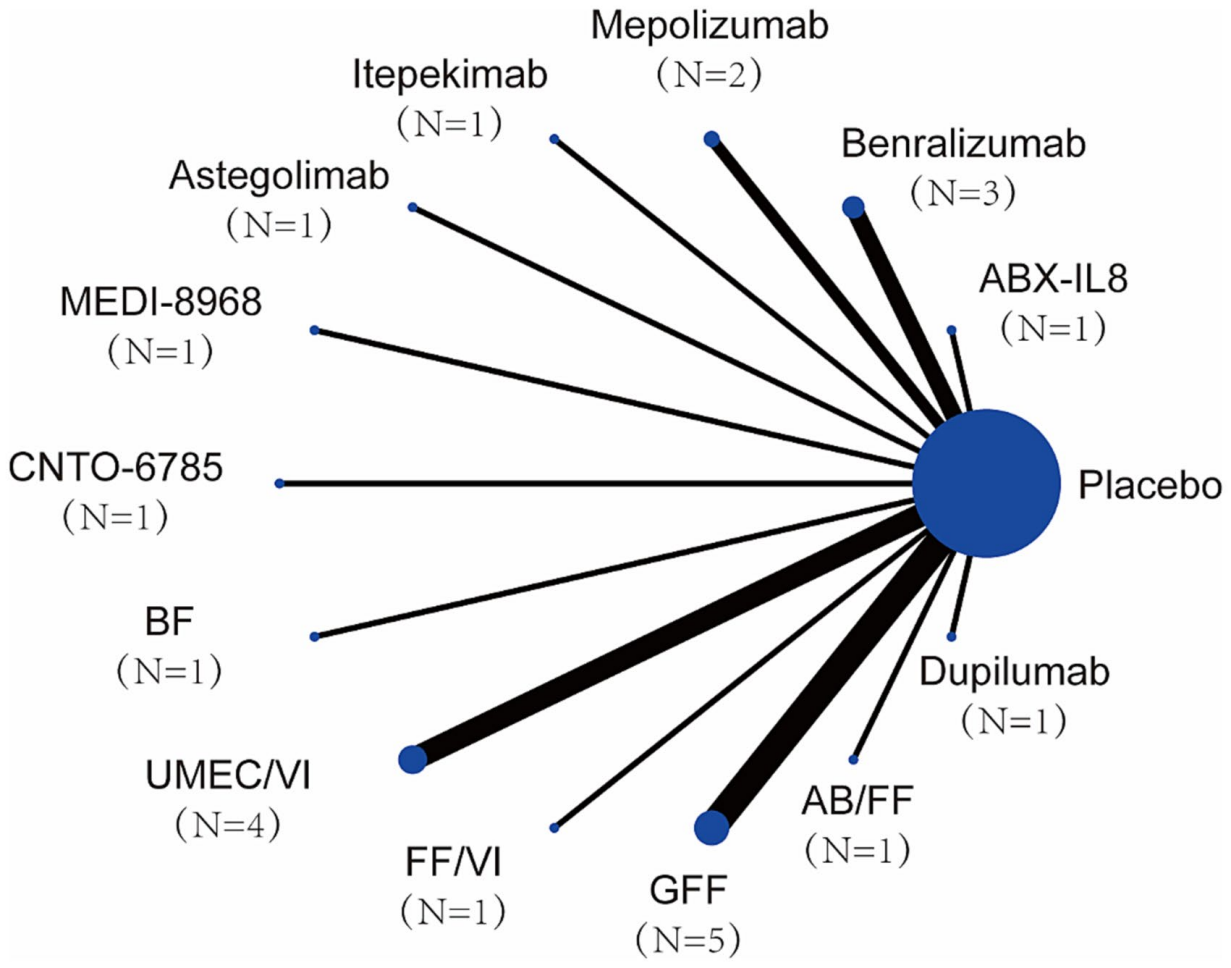
Network map of the study.

In this NMA, we compared the treatment effects and safety of ABX-IL8, Benralizumab, Mepolizumab, Itepekimab, Astegolimab, Dupilumab, MEDI-8968, CNTO-6785, BF, UMEC/VI, GFF, and AB/FF. The network plot is shown in Figure 4, all trials were linked by a common placebo control group and failed to form a closed loop therefore comparisons were made using an indirect model. Among the interventions, UMEC/VI and GFF interventions stand out prominently, in terms of both the number of trials and the number of patients included in the NMA.

### Primary outcome of FEV1 improvement

Twenty-three RCTs involving 20,853 patients compared the efficacy of different mAbs or dual therapies in terms of FEV1 improvement. The results of NMA are presented in Table 2.

**Table 2:** Network meta-analysis comparisons for FEV1 improvement.

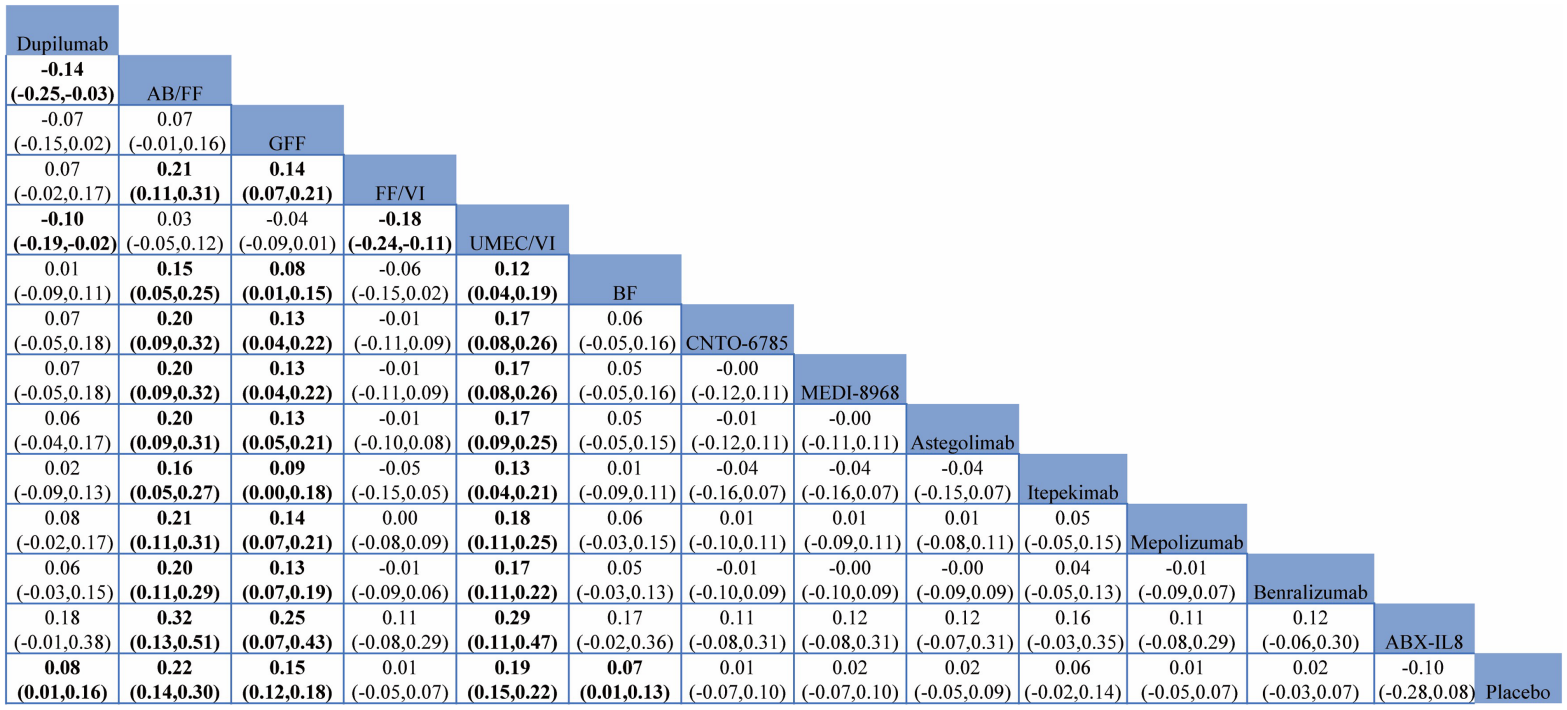

Compared with placebo, Dupilumab (MD = 0.08, 95% CI (0.01, 0.16)), BF (MD = 0.07, 95% CI (0.01, 0.13)), UMEC/VI (MD = 0.19, 95% CI (0.15, 0.22)), GFF (MD = 0.15, 95% CI (0.12, 0.18)), and AB/FF (MD = 0.22, 95% CI (0.14, 0.30)) significantly increased FEV1. Almost all mAbs (ABX-IL8, Benralizumab, Mepolizumab, Itepekimab, Astegolimab, MEDI-8968, CNTO-6785) were inferior to UMEC/VI, AB/FF, and GFF (*p* < 0.05). Dupilumab was inferior to UMEC/VI (MD = −0.10, 95% CI (−0.19, −0.02)) and AB/FF (MD = −0.14, 95% CI (−0.25, −0.03)). When it comes to dual therapies, BF was inferior to UMEC/VI (MD = −0.12, 95% CI (−0.19, −0.04)), GFF (MD = −0.08, 95% CI (−0.15, −0.01)), AB/FF (MD = −0.15, 95% CI (−0.25, −0.05)). FF/VI was inferior to UMEC/VI, (MD = −0.18, 95% CI (−0.24, −0.11)), GFF (MD = −0.14, 95% CI (−0.21, −0.07)), AB/FF (MD = −0.21, 95% CI (−0.31, −0.11)). And no significant differences were observed between AB/FF, UMEC/VI, and GFF. The MD/RR (95%CI) for each study and pooled treatment effect in each comparison were shown in Supplementary Figure S1.

**Figure 5:**
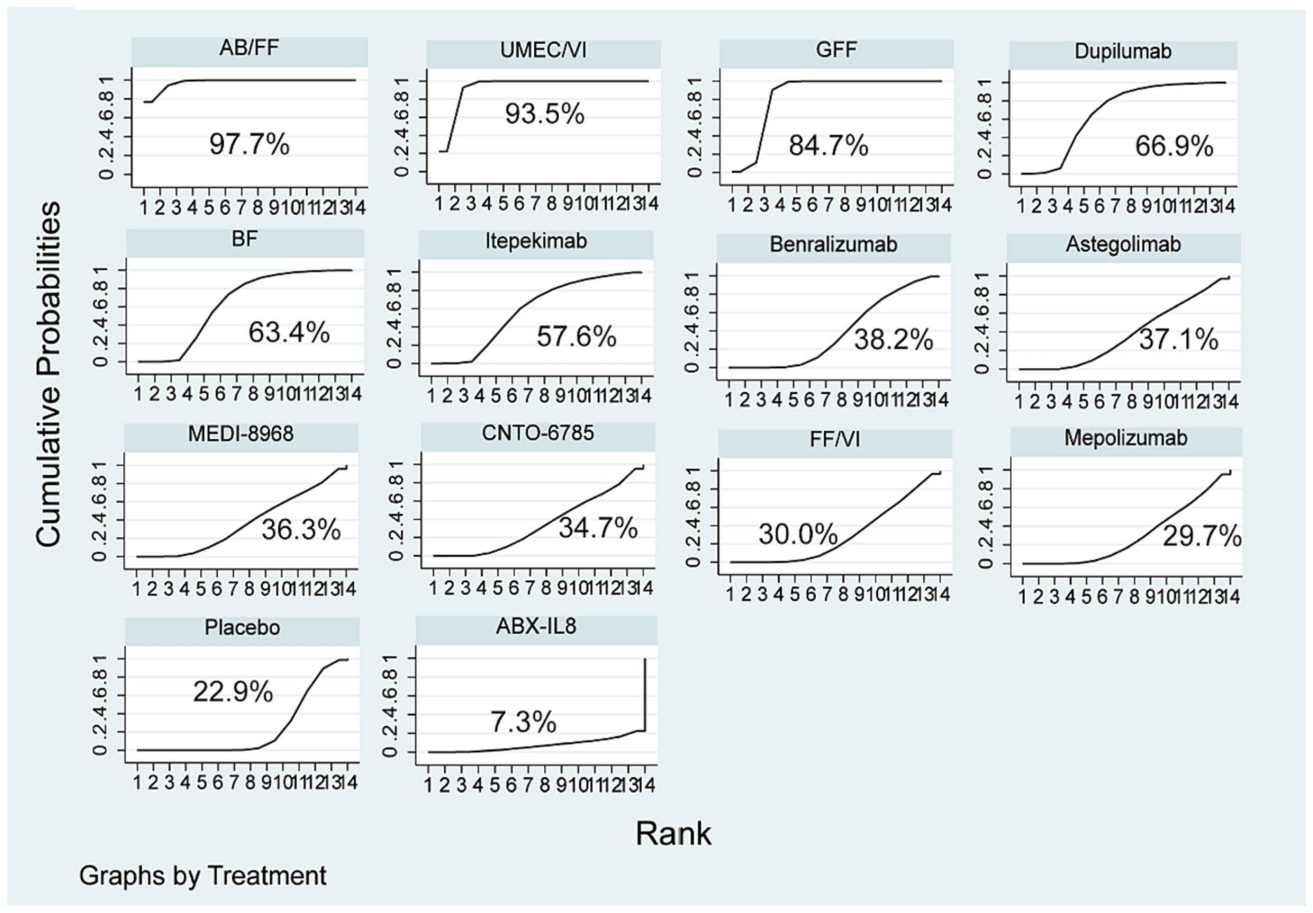
Ranking of treatment strategies based on SUCRA.

The ranking probability based on SUCRA indicated that AB/FF (97.7%) had the highest probability of being the best treatment option for improving FEV1, followed by UMEC/VI (SUCRA 93.5%), and GFF (SUCRA 84.7%). Dupilumab (SUCRA 66.9%) ranked the fourth among all the interventions, while ranked the first among all the mAbs. The details are shown in Figure 5.

### Safety outcomes

For the safety evaluation, this study focused on analyzing the incidence of TEAEs across all the 13 interventions. The incidence of patients with ≥1 TEAEs in conventional dichotomous agents (BF, UMEC/VI, GFF, AB/FF, FF/VI) was between 7.1 and 67.1%. While the incidence of mAbs (ABX-IL8, Benralizumab, Mepolizumab, Itepekimab, Astegolimab, Dupilumab, MEDI-8968, CNTO-6785) was between 61.6 and 88.2% (see Supplementary Table S3).

There was no statistically significant differences in TEAEs rates for any of the interventions compared to placebo (*p* > 0.05) (Supplementary Table S2).

**Figure 6:**
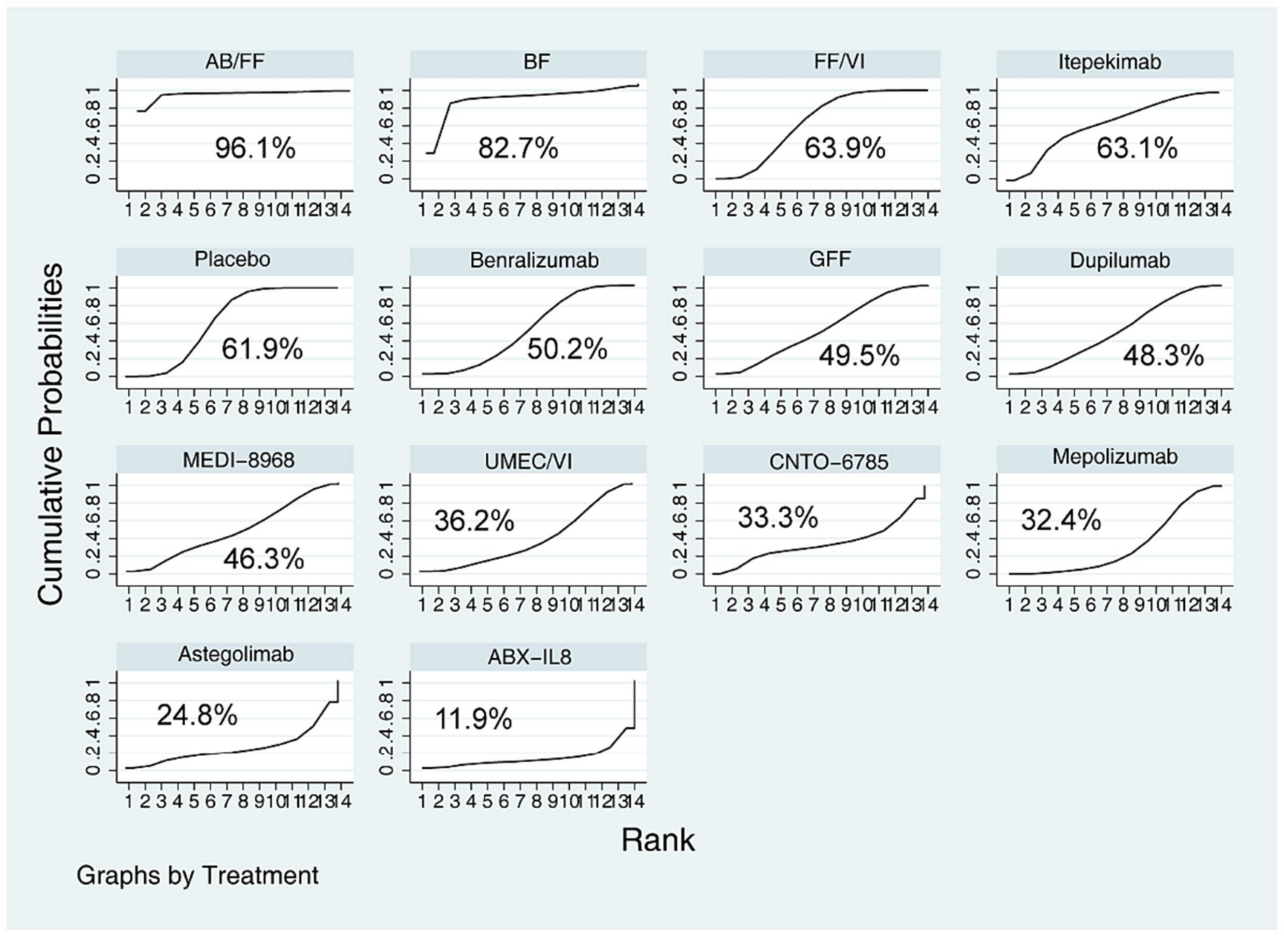
Ranking of safety outcomes based on SUCRA.

AB/FF had a lower incidence of TEAEs than Mepolizumab (RR = 0.56, 95% CI (0.32, 0.99)), Astegolimab (RR = 0.52, 95% CI (0.28, 0.97)), and ABX-IL8 (RR = 0.48, 95% CI (0.26, 0.90)). And with the exception of AB/FF, there was no statistical difference in the incidence of TEAEs between most of the dual agents and mAbs (*p* > 0.05). The ranking probability of safety based on SUCRA showed that Benralizumab, Dupilumab, MEDI-8968, and GFF have consistent safety profiles (50.2% *VS* 48.3% *VS* 46.3% *VS* 49.5%). The SUCRA in Itepekimab, FF/VI, CNTO-6785, and UMEC/VI was 63.1, 63.9, 33.3, 36.2%, respectively (Figure 6). The RR (95%CI) for each study and pooled treatment effect in each comparison were shown in Supplementary Figure S2. AEs reported in each trial were summarized in Supplementary Table S3.

## Discussion

This meta-analysis included 23 RCTs from 20 articles with 20,853 participants and compared the effects of mAbs and some of the traditional duplex preparations (ICS + LABA\LAMA+LABA) on FEV1. We found the data of Interleukin-8(IL-8) antagonist ABX-IL8, IL-5 antagonist Mepolizumab, IL- 5R antagonist Benralizumab, IL-33 antagonist Itepekimab, IL-33/ST2(IL1 receptor-like 1) antagonist Astegolimab, IL-1R1 antagonist MEDI-8968, IL-17A antagonist CNTO-6785, IL-4Rα antagonist Dupilumab. Our results showed that only Dupilumab significantly improved lung function in COPD patients among all mAbs, and it has comparable effects on lung function improvement as compared to GFF, BF, FF/VI. Therefore, from the perspective of FEV1 improvement, COPD patients can benefit from Dupilumab as a treatment option.

The results of Benralizumab and Itepekimab in the present study were consistent with those of the study conducted by Wu et al. (34). However, Wu et al. did not include as extensive a body of literature as the present study. In previous studies, Benralizumab performed well in all mAbs against COPD (35). Benralizumab, targeting and blocking the action of IL-5 receptor alpha (IL-5Rα), has received a great deal of attention after previous studies suggested that targeting the IL-5-activated pathway may be beneficial for COPD patients (36, 37). IL-5 is a cytokine that plays a crucial role in the activation and survival of eosinophils, a type of white blood cell involved in the immune response (38). By blocking IL-5Rα, Benralizumab reduces the number of eosinophils in the blood and airways, thereby reducing airway inflammation (36, 39). The meta-analysis by Paola et al. also showed that Benralizumab is very promising for development (35). However, according to the results of this study, increasing the latest research results of Benralizumab (NCT02138916 and NCT02155660), it did not show enough advantage in terms of pulmonary function improvement. Nevertheless, it is worth stating that this study could not rule out a potential benefit of benralizumab in COPD patients with high eosinophil count. Therefore, future studies focusing on the effects of Benralizumab/Mepolizumab on lung function in eosinophilic COPD subgroup are needed.

In this study, Dupilumab showed better developmental promise as far as lung function improvement is concerned (40). Dupilumab is a fully human monoclonal antibody that binds IL-4Rα and inhibits signaling of both IL-4 and IL-13 (33), which plays a central role in the inflammatory processes. Moreover, IL-5/IL-5Rα, Immunoglobulin E (IgE) and Janus Kinase(JAK1/2) are all downstream of the IL-4/IL-13 signaling pathway, whereas by inhibiting IL-4Rα it is possible to block the IL-4/IL-13 signaling pathway from upstream, and thus inhibit T helper 2 cell(Th2)-mediated type II inflammation (41). Based on the RCT results of Dupilumab, in addition to improved lung function, patients treated with Dupilumab had fewer exacerbations, better quality of life, and fewer respiratory symptoms than those treated with placebo, which is a very promising application. However, it is true that in this study, there is no way to avoid the impact of data imbalance on the results, especially since there is currently only one RCT result of Dupilumab. Like Benralizumab, which demonstrated favourable clinical outcomes in the 2014 study but failed to achieve the desired results in a large phase 3 trial with an expanded sample size at a later stage, Mepolizumab suffers from similar problems. Nevertheless, it is interesting to note that the upfront benefits of both Dupilumab and Mepolizumab were based on small sample sizes (*N* < 100), and in the Dupilumab study, the sample size with good clinical endpoints was 939, so Dupilumab remains a promising mAbs for the treatment of COPD. Therefore, it is crucial to closely monitor the ongoing Phase 3 study of Dupilumab (NCT04456673) for further insights in the long term. Furthermore, although in this study, ABX-IL8, Mepolizumab, Itepekimab, Astegolimab, MEDI-8968, and CNTO-6785 do not appear to have a significant clinical impact on improving lung function in COPD, however, as studies are conducted and deepened, it may not be impossible to demonstrate the use of mAbs in patients with other COPD subtypes.

There are limitations to this study. Firstly, the mAbs studies are generally administered on top of bronchodilators, so the comparison versus dual bronchodilator effects here is somewhat flawed. But this study was an attempt to compare the difference in the degree of improvement in FEV1 between the mAbs in its normal state of use and the dual bronchodilator, which still showed some degree of improvement in FEV1 from the performance of the Dupilumab. Second, there was some inconsistency in the inclusion and exclusion criteria concerning the study period, with certain studies encompassing longer durations while others had shorter ones. This discrepancy could potentially influence the study outcomes. Third, exacerbation rates as well as mortality were the end points for COPD. Solely assessing the improvement in FEV1 in this study may be limiting because FEV1 alone may not provide the most comprehensive evaluation for the treatment of chronic airway conditions. Finally, the unbalanced number of trials may affect the reliability of the findings, especially as only 1 study was included for many drugs, for example, the data of Dupilumab only from one RCT (NCT03930732), whereas those for Mepolizumab (NCT01463644, NCT02105961, NCT02105948) and Benralizumab (NCT01227278, NCT02138916, NCT02155660) were extracted from three RCTs, respectively. Therefore, future updates on this topic will be needed with more studies available.

## Conclusion

In conclusion, of the 8 mABs (ABX-IL8, Benralizumab, Mepolizumab, Itepekimab, Astegolimab, Dupilumab, MEDI-8968, CNTO-6785) included in the NMA, only Dupilumab significantly improved lung function in COPD patients, and it has comparable effects as compared to GFF, BF, FF/VI. However, additional studies are required to confirm the findings of this study, and the ongoing Phase 3 study of Dupilumab (NCT04456673) deserves continued attention.

## Data availability statement

The original contributions presented in the study are included in the article/Supplementary material, further inquiries can be directed to the corresponding authors.

## Author contributions

YX: Writing – original draft. J-qH: Writing – original draft. H-lT: Writing – review & editing. Z-xZ: Writing – review & editing. L-hL: Writing – review & editing.

## References

[1] Agustí A Celli BR Criner GJ Halpin D Anzueto A Barnes P . Global initiative for chronic obstructive lung disease 2023 report: GOLD executive summary. Eur Respir J. (2023) 61:2300239. doi: 10.1183/13993003.00239-2023, PMID: 36858443 PMC10066569

[2] Adeloye D Song P Zhu Y Campbell H Sheikh A Rudan I. Global, regional, and national prevalence of, and risk factors for, chronic obstructive pulmonary disease (COPD) in 2019: a systematic review and modelling analysis. Lancet Respir Med. (2022) 10:447–58. doi: 10.1016/S2213-2600(21)00511-7, PMID: 35279265 PMC9050565

[3] Fei F J Siegert R Zhang X Gao W Koffman J. Symptom clusters, associated factors and health-related quality of life in patients with chronic obstructive pulmonary disease: A structural equation modelling analysis. J Clin Nurs. (2023) 32:298–310. doi: 10.1111/jocn.16234 35098602 PMC10078635

[4] Cazzola M Molimard M. The scientific rationale for combining long-acting beta2-agonists and muscarinic antagonists in COPD. Pulm Pharmacol Ther. (2010) 23:257–67. doi: 10.1016/j.pupt.2010.03.003, PMID: 20381630

[5] Farne HA Cates CJ. Long-acting beta2-agonist in addition to tiotropium versus either tiotropium or long-acting beta2-agonist alone for chronic obstructive pulmonary disease. Cochrane Database Syst Rev. (2015) 2015:CD008989. doi: 10.1002/14651858.CD008989.pub3 PMC12047630 26490945

[6] Nannini LJ Lasserson TJ Poole P. Combined corticosteroid and long-acting beta(2)-agonist in one inhaler versus long-acting beta(2)-agonists for chronic obstructive pulmonary disease. Cochrane Database Syst Rev. (2012) 2012:CD006829. doi: 10.1002/14651858.CD006829.pub2 22972099 PMC4170910

[7] Nannini LJ Poole P Milan SJ Kesterton A. Combined corticosteroid and long-acting beta(2)-agonist in one inhaler versus inhaled corticosteroids alone for chronic obstructive pulmonary disease. Cochrane Database Syst Rev. (2013) 2013:CD006826. doi: 10.1002/14651858.CD006826.pub2 23990350 PMC6486274

[8] Gross NJ Barnes PJ. New therapies for asthma and chronic obstructive pulmonary disease. Am J Respir Crit Care Med. (2017) 195:159–66. doi: 10.1164/rccm.201610-2074PP 27922751

[9] Barnes PJ . Corticosteroid resistance in patients with asthma and chronic obstructive pulmonary disease. J Allergy Clin Immunol. (2013) 131:636–45. doi: 10.1016/j.jaci.2012.12.1564 23360759

[10] Cazzola M Page CP Calzetta L Matera MG. Emerging anti-inflammatory strategies for COPD. Eur Respir J. (2012) 40:724–41. doi: 10.1183/09031936.00213711 22496331

[11] Matera MG Page C Rogliani P Calzetta L Cazzola M. Therapeutic monoclonal antibodies for the treatment of chronic obstructive pulmonary disease. Drugs. (2016) 76:1257–70. doi: 10.1007/s40265-016-0625-9 27506851

[12] Higgins JPT Thomas J Chandler J Cumpston M Li T Page MJ . Cochrane Handbook for Systematic Reviews of Interventions version 6.4 Cochrane. (2023). Available from: www.training.cochrane.org/handbook.

[13] Sterne JAC Savović J Page MJ Elbers RG Blencowe NS Boutron I . RoB 2: a revised tool for assessing risk of bias in randomised trials. BMJ. (2019) 366:l4898. doi: 10.1136/bmj.l4898 31462531

[14] Koopman M Franssen FME Gaffron S Watz H Troosters T Garcia-Aymerich J . Differential outcomes following 4 weeks of Aclidinium/formoterol in patients with COPD: a reanalysis of the ACTIVATE study. Int J Chron Obstruct Pulmon Dis. (2022) 17:517–33. doi: 10.2147/COPD.S308600, PMID: 35342289 PMC8943652

[15] Lipworth BJ Collier DJ Gon Y Zhong N Nishi K Chen R . Improved lung function and patient-reported outcomes with co-suspension delivery technology glycopyrrolate/formoterol fumarate metered dose inhaler in COPD: a randomized phase III study conducted in Asia, Europe, and the USA. Int J Chron Obstruct Pulmon Dis. (2018) 13:2969–84. doi: 10.2147/COPD.S171835, PMID: 30310273 PMC6167125

[16] Reisner C Gottschlich G Fakih F Koser A Krainson J Delacruz L . 24-h bronchodilation and inspiratory capacity improvements with glycopyrrolate/formoterol fumarate via co-suspension delivery technology in COPD. Respir Res. (2017) 18:157. doi: 10.1186/s12931-017-0636-4 28821260 PMC5563048

[17] Martinez FJ Rabe KF Ferguson GT Fabbri LM Rennard S Feldman GJ . Efficacy and safety of Glycopyrrolate/formoterol metered dose inhaler formulated using co-suspension delivery Technology in Patients with COPD. Chest. (2017) 151:340–57. doi: 10.1016/j.chest.2016.11.028, PMID: 27916620

[18] Vestbo J Anderson JA Brook RD Calverley PM Celli BR Crim C . Fluticasone furoate and vilanterol and survival in chronic obstructive pulmonary disease with heightened cardiovascular risk (SUMMIT): a double-blind randomised controlled trial. Lancet. (2016) 387:1817–26. doi: 10.1016/S0140-6736(16)30069-1 27203508

[19] Zheng J Zhong N Newlands A Church A Goh AH. Efficacy and safety of once-daily inhaled umeclidinium/vilanterol in Asian patients with COPD: results from a randomized, placebo-controlled study. Int J Chron Obstruct Pulmon Dis. (2015) 10:1753–67. doi: 10.2147/COPD.S81053, PMID: 26366068 PMC4562726

[20] Maltais F Singh S Donald AC Crater G Church A Goh AH . Effects of a combination of umeclidinium/vilanterol on exercise endurance in patients with chronic obstructive pulmonary disease: two randomized, double-blind clinical trials. Ther Adv Respir Dis. (2014) 8:169–81. doi: 10.1177/1753465814559209, PMID: 25452426

[21] Celli B Crater G Kilbride S Mehta R Tabberer M Kalberg CJ . Once-daily umeclidinium/vilanterol 125/25 mcg in COPD: a randomized, controlled study. Chest. (2014) 145:981–91. doi: 10.1378/chest.13-1579 24385182

[22] Donohue JF Maleki-Yazdi MR Kilbride S Mehta R Kalberg C Church A. Efficacy and safety of once-daily umeclidinium/vilanterol 62.5/25 mcg in COPD. Respir Med. (2013) 107:1538–46. doi: 10.1016/j.rmed.2013.06.001, PMID: 23830094

[23] Tashkin DP Doherty DE Kerwin E Matiz-Bueno CE Knorr B Shekar T . “Efficacy and safety of budesonide and formoterol in one pressurized metered-dose inhaler in patients with moderate to very severe chronic obstructive pulmonary disease: results of a 6-month randomized clinical trial.” Drugs. (2008) 14:1975–2000. doi: 10.2165/00003495-200868140-00004 18778120

[24] Mahler DA Huang S Tabrizi M Bell GM. Efficacy and safety of a monoclonal antibody recognizing interleukin-8 in COPD: a pilot study. Chest. (2004) 126:926–34. doi: 10.1378/chest.126.3.926, PMID: 15364775

[25] Brightling CE Bleecker ER Panettieri RA Jr Bafadhel M She D Ward CK . Benralizumab for chronic obstructive pulmonary disease and sputum eosinophilia: a randomised, double-blind, placebo-controlled, phase 2a study. The lancet. Respir Med. (2014) 2:891–901. doi: 10.1016/S2213-2600(14)70187-0 PMC5082845 25208464

[26] Criner GJ Celli BR Brightling CE Agusti A Papi A Singh D . Benralizumab for the prevention of COPD exacerbations. N Engl J Med. (2019) 381:1023–34. doi: 10.1056/NEJMoa1905248, PMID: 31112385

[27] Rabe KF Celli BR Wechsler ME Abdulai RM Luo X Boomsma MM . Safety and efficacy of itepekimab in patients with moderate-to-severe COPD: a genetic association study and randomised, double-blind, phase 2a trial. Lancet Respir Med. (2021) 9:1288–98. doi: 10.1016/S2213-2600(21)00167-3, PMID: 34302758

[28] Pavord ID Chapman KR Bafadhel M Sciurba FC Bradford ES Schweiker Harris S . Mepolizumab for eosinophil-associated COPD: analysis of METREX and METREO. Int J Chron Obstruct Pulmon Dis. (2021) 16:1755–70. doi: 10.2147/COPD.S294333, PMID: 34163157 PMC8215850

[29] Yousuf AJ Mohammed S Carr L Yavari Ramsheh M Micieli C Mistry V . Astegolimab, an anti-ST2, in chronic obstructive pulmonary disease (COPD-ST2OP): a phase 2a, placebo-controlled trial. Lancet Respir Med. (2022) 10:469–77. doi: 10.1016/S2213-2600(21)00556-7, PMID: 35339234

[30] Eich A Urban V Jutel M Vlcek J Shim JJ Trofimov VI . A randomized, placebo-controlled phase 2 trial of CNTO 6785 in chronic obstructive pulmonary disease. COPD. (2017) 14:476–83. doi: 10.1080/15412555.2017.1335697, PMID: 28753067

[31] Calverley PMA Sethi S Dawson M Ward CK Finch DK Penney M . A randomised, placebo-controlled trial of anti-interleukin-1 receptor 1 monoclonal antibody MEDI8968 in chronic obstructive pulmonary disease. Respir Res. (2017) 18:153. doi: 10.1186/s12931-017-0633-7, PMID: 28793896 PMC5551010

[32] Dasgupta A Kjarsgaard M Capaldi D Radford K Aleman F Boylan C . A pilot randomised clinical trial of mepolizumab in COPD with eosinophilic bronchitis. Eur Respir J. (2017) 49:1602486. doi: 10.1183/13993003.02486-2016, PMID: 28298405

[33] Bhatt SP Rabe KF Hanania NA Vogelmeier CF Cole J Bafadhel M . Dupilumab for COPD with type 2 inflammation indicated by eosinophil counts. N Engl J Med. (2023) 389:205–14. doi: 10.1056/NEJMoa2303951, PMID: 37272521

[34] Wu Y Huang M Zhong J Lu Y Gan K Yang R . The clinical efficacy of type 2 monoclonal antibodies in eosinophil-associated chronic airway diseases: a meta-analysis. Front Immunol. (2023) 14:1089710. doi: 10.3389/fimmu.2023.1089710, PMID: 37114057 PMC10126252

[35] Rogliani P Matera MG Puxeddu E Mantero M Blasi F Cazzola M . Emerging biological therapies for treating chronic obstructive pulmonary disease: a pairwise and network meta-analysis. Pulm Pharmacol Ther. (2018) 50:28–37. doi: 10.1016/j.pupt.2018.03.004, PMID: 29609004

[36] Bel EH Ten Brinke A. New anti-eosinophil drugs for asthma and COPD: targeting the trait! Chest. (2017) 152:1276–82. doi: 10.1016/j.chest.2017.05.019, PMID: 28583618

[37] Kouro T Takatsu K. IL-5- and eosinophil-mediated inflammation: from discovery to therapy. Int Immunol. (2009) 21:1303–9. doi: 10.1093/intimm/dxp102, PMID: 19819937

[38] Narendra DK Hanania NA. Targeting IL-5 in COPD. Int J Chron Obstruct Pulmon Dis. (2019) 14:1045–51. doi: 10.2147/COPD.S155306, PMID: 31190789 PMC6529620

[39] Markham A . Benralizumab: First Global Approval. Drugs. (2018) 78:505–11. doi: 10.1007/s40265-018-0876-8, PMID: 29464664

[40] Kodaka N Nakano C Oshio T Hirouchi T Yamada Y Matsuse H. Effects of Dupilumab for asthma-chronic obstructive pulmonary disease overlap. Iran J Allergy Asthma Immunol. (2023) 22:212–6. doi: 10.18502/ijaai.v22i2.12683, PMID: 37496415

[41] Harb H Chatila TA. Mechanisms of Dupilumab. Clin Exp Allergy. (2020) 50:5–14. doi: 10.1111/cea.13491, PMID: 31505066 PMC6930967

