# Supplementary material for: Network meta-analysis of the efficacy and safety of monoclonal antibodies and traditional conventional dichotomous agents for chronic obstructive pulmonary disease
Source: Front Med (Lausanne). 2024 Feb 13;11:1334442. doi: 10.3389/fmed.2024.1334442 (PMC10898352; doi:10.3389/fmed.2024.1334442)
Supplement: Supplementary file 1 [file Data_Sheet_1.docx]

Supplementary material

Table S1 Search strategies of each database

| 1. Search strategies of PubMed |
| --- |
| #1. "pulmonary disease, chronic obstructive"[MeSH Terms]  #2. "chronic obstructive lung disease"[Title/Abstract] OR "chronic obstructive pulmonary diseases"[Title/Abstract] OR "COAD"[Title/Abstract] OR "COPD"[Title/Abstract] OR "COBD"[Title/Abstract] OR "chronic obstructive airway disease"[Title/Abstract] OR "chronic obstructive pulmonary disease"[Title/Abstract] OR "airflow obstruction chronic"[Title/Abstract] OR "airflow obstructions chronic"[Title/Abstract] OR "chronic airflow obstructions"[Title/Abstract] OR "chronic airflow obstruction"[Title/Abstract] OR "chronic obstructive lung disease"[Title/Abstract]  #3. #1 OR #2  #4. "mepolizumab"[Supplementary Concept]  #5. "Bosatria"[Title/Abstract] OR "SB-240563"[Title/Abstract] OR "SB240563"[Title/Abstract] OR "Nucala"[Title/Abstract]  #6. #4 OR #5  #7. "reslizumab"[Supplementary Concept]  #8. "Cinquil"[Title/Abstract] OR "Cinqair"[Title/Abstract] OR "SCH-55700"[Title/Abstract] OR "SCH55700"[Title/Abstract] OR "SCH-55700"[Title/Abstract] OR "DCP-835"[Title/Abstract] OR "DCP-835"[Title/Abstract] OR "reslizumab*"[Title/Abstract]  #9. #7 OR #8  #10. "benralizumab"[Supplementary Concept]  #11. "benralizumab*"[Title/Abstract] OR "MEDI-563"[Title/Abstract] OR "MEDI-563"[Title/Abstract] OR "Fasenra"[Title/Abstract] OR "BIW-8405"[Title/Abstract]  #12. #10 OR #11  #13. "canakinumab"[Supplementary Concept]  #14. "Ilaris"[Title/Abstract] OR "ACZ-885"[Title/Abstract] OR "ACZ885"[Title/Abstract] OR "canakinumab*"[Title/Abstract] OR "immunoglobulin g1"[Title/Abstract] OR ("anti-human"[All Fields] AND "interleukin 1beta"[Title/Abstract]) OR (("anti-human"[All Fields] AND ("clone cells"[MeSH Terms] OR ("clone"[All Fields] AND "cells"[All Fields]) OR "clone cells"[All Fields] OR "clone"[All Fields] OR "cloned"[All Fields] OR "clones"[All Fields] OR "clonings"[All Fields] OR "clone s"[All Fields] OR "cloning, organism"[MeSH Terms] OR ("cloning"[All Fields] AND "organism"[All Fields]) OR "organism cloning"[All Fields] OR "cloning"[All Fields]) AND ("canakinumab"[Supplementary Concept] OR "canakinumab"[All Fields] OR "ACZ885"[All Fields])) AND "heavy chain v region"[Title/Abstract])  #15. #13 OR #14  #16. "ABX-Interleukin 8"[Supplementary Concept]  #17. "abx interleukin 8"[Supplementary Concept] OR "abx interleukin 8"[All Fields]  #18. #16 OR #17  #19. "Infliximab"[MeSH Terms]  #20. "mab ca2"[Title/Abstract] OR "monoclonal antibody ca2"[Title/Abstract] OR (("antibodie"[All Fields] OR "antibodies"[MeSH Terms] OR "antibodies"[All Fields] OR "antibody s"[All Fields] OR "antibodys"[All Fields] OR "immunoglobulins"[MeSH Terms] OR "immunoglobulins"[All Fields] OR "Antibody"[All Fields]) AND "ca2 monoclonal"[Title/Abstract]) OR "ca2 monoclonal antibody"[Title/Abstract] OR "Infliximab-dyyb"[Title/Abstract] OR "Infliximab-dyyb"[Title/Abstract] OR "Inflectra"[Title/Abstract] OR "Remicade"[Title/Abstract] OR "Infliximab-abda"[Title/Abstract] OR "Infliximab-abda"[Title/Abstract] OR "Renflexis"[Title/Abstract] OR "infliximab*"[Title/Abstract]  #21. #19 OR #20  #22. "Etanercept"[MeSH Terms]  #23. "tnfr fc fusion protein"[Title/Abstract] OR "fusion protein tnfr fc"[Title/Abstract] OR "tnfr fc fusion protein"[Title/Abstract] OR "TNR-001"[Title/Abstract] OR "TNR-001"[Title/Abstract] OR (("trinitrotoluene"[MeSH Terms] OR "trinitrotoluene"[All Fields] OR "tnt"[All Fields]) AND "receptor fusion protein"[Title/Abstract]) OR "Etanercept-szzs"[Title/Abstract] OR "tnf receptor type ii igg fusion protein"[Title/Abstract] OR "tnf receptor type ii igg fusion protein"[Title/Abstract] OR "Erelzi"[Title/Abstract] OR "recombinant human dimeric tnf receptor type ii igg fusion protein"[Title/Abstract] OR "recombinant human dimeric tnf receptor type ii igg fusion protein"[Title/Abstract] OR "Enbrel"[Title/Abstract]  #24. #22 OR #23  #25. "itepekimab"[Supplementary Concept]  #26. (((((REGN-3500[Title/Abstract]) OR (REGN3500[Title/Abstract])) OR (SAR-440340[Title/Abstract])) OR (SAR440340[Title/Abstract])) ("itepekimab" [Supplementary Concept])) OR (((((REGN-3500[Title/Abstract]) OR (REGN3500[Title/Abstract])) OR (SAR-440340[Title/Abstract])) OR (SAR440340[Title/Abstract])) OR (Itepekimab*[Title/Abstract]))  #27. #25 OR #26  #28. "astegolimab"[Supplementary Concept]  #29. "MSTT1041A"[Title/Abstract] OR "astegolimab*"[Title/Abstract]  #30. #28 OR #29  #31. "lebrikizumab"[Supplementary Concept] OR "lebrikizumab*"[Title/Abstract]  #32. "CNTO6785"[Supplementary Concept]  #33. ((((AMG 282[Title/Abstract]) OR (Tozorakimab*[Title/Abstract])) OR (Tozorakimab[Title/Abstract])) OR (MEDI3506[Title/Abstract])) OR (MEDI8986[Title/Abstract])  #34. "dupilumab"[Supplementary Concept] OR ("SAR231893"[Title/Abstract] OR "SAR-231893"[Title/Abstract] OR "Dupixent"[Title/Abstract] OR "REGN668"[Title/Abstract] OR "REGN-668"[Title/Abstract])  #35. "Randomized Controlled Trial"[Publication Type]  #36. (("randomized controlled trial"[Publication Type] OR "controlled clinical trial"[Publication Type] OR "randomized"[Title/Abstract] OR "placebo"[Title/Abstract] OR "drug therapy"[MeSH Subheading] OR "randomly"[Title/Abstract] OR "trial"[Title/Abstract] OR "groups"[Title/Abstract]) NOT "animals"[MeSH Terms]) NOT "humans"[MeSH Terms]  #37. #35 OR #36  #38. #6 OR #9 OR #12 OR #15 OR #18 OR #21 OR #24 OR #27 OR #30 OR #31 OR #32 OR #33 OR #34  #39. "Formoterol Fumarate"[MeSH Terms]  #40. ((((((3-Formylamino-4-hydroxy-alpha-(N-1-methyl-2-p-methoxyphenethylaminomethyl)benzylalcohol.hemifumarate[Title/Abstract]) OR (BD 40A[Title/Abstract])) OR (Formoterol[Title/Abstract])) OR (Eformoterol[Title/Abstract])) OR (Oxis[Title/Abstract])) OR (Arformoterol[Title/Abstract])) OR (Foradil[Title/Abstract])  #41. #39 OR #40  #42. "aclidinium bromide"[Supplementary Concept]  #43. ((3R)-3-((hydroxy(di-2-thienyl)acetyl)oxy)-1-(3-phenoxypropyl)-1-azoniabicyclo(2.2.2)octane bromide[Title/Abstract]) OR (aclidinium*[Title/Abstract])  #44. #42 OR #43  #45. "Glycopyrrolate"[Mesh]  #46. ((((Glycopyrronium Bromide[Title/Abstract]) OR (Glycopyrronium[Title/Abstract])) OR (Pyrrolidinium, 3-((cyclopentylhydroxyphenylacetyl)oxy)-1,1-dimethyl-, bromide[Title/Abstract])) OR (NVA237[Title/Abstract])) OR (glycopyrronium*[Title/Abstract])  #47. #45 OR #46  #48. "indacaterol" [Supplementary Concept]  #49. ((((indacaterol*[Title/Abstract]) OR (5-(2-(5,6-diethylindan-2-ylamino)-1-hydroxyethyl)-8-hydroxy-1H-quinolin-2-one[Title/Abstract])) OR (QAB-149[Title/Abstract])) OR (arcapta neohaler[Title/Abstract])) OR (Onbrez[Title/Abstract])  #50. #48 OR #49  #51. "vilanterol" [Supplementary Concept]  #52. (vilanterol*[Title/Abstract]) OR (4-(2-((6-((2-(((2,6-dichlorophenyl)methyl)oxy)ethyl)oxy)hexyl)amino)-1-hydroxyethyl)-2-(hydroxymethyl)phenol[Title/Abstract])  #53. #51 OR #52  #54. ("GSK573719" [Supplementary Concept]) OR ((umeclidinium*[Title/Abstract]) OR (umeclidinium[Title/Abstract]))  #55. ("olodaterol" [Supplementary Concept]) OR (((((Olodaterol[Title/Abstract]) OR (Olodaterol*[Title/Abstract])) OR (6-hydroxy-8-(1-hydroxy-2-((2-(4-methoxyphenyl)-1,1-dimethylethyl)amino)ethyl)-2H-1,4-benzoxazin-3(4H)-one[Title/Abstract])) OR (BI 1744 CL[Title/Abstract])) OR (Striverdi Respimat[Title/Abstract]))  #56. ("Tiotropium Bromide"[Mesh]) OR ((((((Tiotropium Bromide[Title/Abstract]) OR (7-((hydroxybis(2-thienyl)acetyl)oxy)-9,9-dimethyl-3-oxa-9-azoniatricyclo(3.3.1.0(2,4))nonane bromide[Title/Abstract])) OR (Tiotropium[Title/Abstract])) OR (Tiotropium*[Title/Abstract])) OR (Spiriva[Title/Abstract])) OR (BA 679 BR[Title/Abstract]))  #57. ("Budesonide"[Mesh]) OR ((((((((budesonide[Title/Abstract]) OR (budesonide*[Title/Abstract])) OR (Rhinocort[Title/Abstract])) OR (Horacort[Title/Abstract])) OR (Budesonide, (R)-Isomer[Title/Abstract])) OR (Budesonide, (S)-Isomer[Title/Abstract])) OR (Pulmicort[Title/Abstract])) OR (Tarpeyo[Title/Abstract]))  #58. #41 OR #44 OR #47 OR #50 OR #53 OR #54 OR #55 OR #56 OR #57  #59. #38 OR #58  #60 #3 AND #37 AND #59 |
| 1. Search strategies of Embase |
| #1. 'chronic obstructive lung disease'/exp  #2. 'chronic airflow obstruction':ab,ti OR 'chronic airway obstruction':ab,ti OR 'chronic obstructive bronchopulmonary disease':ab,ti OR 'chronic obstructive lung disorder':ab,ti OR 'chronic obstructive lung disease':ab,ti OR 'chronic obstructive airway disease':ab,ti OR 'chronic obstructive pulmonary disease':ab,ti OR 'chronic obstructive pulmonary disorder':ab,ti OR 'chronic obstructive respiratory disease':ab,ti OR 'chronic pulmonary obstructive disease':ab,ti OR 'chronic pulmonary obstructive disorder':ab,ti OR copd:ab,ti OR coad:ab,ti OR cobd:ab,ti OR 'lung chronic obstructive disease':ab,ti OR 'airflow obstruction, chronic':ab,ti OR 'airflow obstructions, chronic':ab,ti OR 'chronic airflow obstructions':ab,ti OR 'obstructive chronic lung disease':ab,ti OR 'obstructive chronic pulmonary disease':ab,ti OR 'lung disease, chronic obstructive':ab,ti OR 'obstructive lung disease, chronic':ab,ti OR 'pulmonary disease, chronic obstructive':ab,ti OR 'pulmonary disorder, chronic obstructive':ab,ti  #3. #1 OR #2  #4. 'mepolizumab'/exp  #5. bosatria:ab,ti OR 'sb 240563':ab,ti OR sb240563:ab,ti OR nucala:ab,ti OR mepolizumab*:ab,ti  #6. #4 OR #5  #7. 'reslizumab'/exp  #8. cinquil:ab,ti OR cinqair:ab,ti OR sch55700:ab,ti OR 'sch 55700':ab,ti OR 'cep 38072':ab,ti OR cep38072:ab,ti OR dcp835:ab,ti OR 'dcp 835':ab,ti OR reslizumab*:ab,ti  #9. #7 OR #8  #10. 'benralizumab'/exp  #11. benralizumab*:ab,ti OR 'medi 563':ab,ti OR fasenra:ab,ti OR 'biw 8405':ab,ti  #12. #10 OR #11  #13. 'canakinumab'/exp  #14. ilaris:ab,ti OR 'acz 885':ab,ti OR acz885:ab,ti OR canakinumab*:ab,ti OR 'immunoglobulin g1':ab,ti OR 'anti-human interleukin 1beta':ab,ti OR 'anti-human clone acz885 heavy chain v region':ab,ti OR 'cmab 816':ab,ti OR cmab816:ab,ti  #15. #13 OR #14  #16. 'abx il8'/exp  #17. 'abx-interleukin 8':ab,ti OR 'abx-il8 mab':ab,ti  #18. #16 OR #17  #19. 'infliximab'/exp  #20. 'abp 710':ab,ti OR abp710:ab,ti OR avakine:ab,ti OR avsola:ab,ti OR 'bcd 055':ab,ti OR bcd055:ab,ti OR 'bow 015':ab,ti OR bow015:ab,ti OR 'cmab 008':ab,ti OR cmab008:ab,ti OR 'ct p13':ab,ti OR ctp13:ab,ti OR flixabi:ab,ti OR 'gb 242':ab,ti OR gb242:ab,ti OR 'gp 1111':ab,ti OR gp1111:ab,ti OR inflectra:ab,ti OR 'infliximab abda':ab,ti OR 'infliximab axxq':ab,ti OR 'infliximab dyyb':ab,ti OR 'infliximab qbtx':ab,ti OR ixifi:ab,ti OR 'pf 06438179':ab,ti OR 'pf 6438179':ab,ti OR pf06438179:ab,ti OR pf6438179:ab,ti OR remicade:ab,ti OR remsima:ab,ti OR renflexis:ab,ti OR revellex:ab,ti OR 'sti 002':ab,ti OR sti002:ab,ti OR 'ta 650':ab,ti OR ta650:ab,ti OR 'zessly mab ca2':ab,ti OR 'monoclonal antibody ca2':ab,ti OR 'antibody ca2, monoclonal':ab,ti OR 'ca2, monoclonal antibody':ab,ti OR infliximab*:ab,ti  #21. #19 OR #20  #22. 'etanercept'/exp  #23. 'avent; benepali':ab,ti OR brenzys:ab,ti OR 'chs 0214':ab,ti OR chs0214:ab,ti OR 'dwp 422':ab,ti OR dwp422:ab,ti OR embrel:ab,ti OR enbrel:ab,ti OR enerceptan:ab,ti OR 'enia 11':ab,ti OR enia11:ab,ti OR erelzi:ab,ti OR etacept:ab,ti OR etanar:ab,ti OR 'etanercept ykro':ab,ti OR eticovo:ab,ti OR 'gp 2015':ab,ti OR 'gp 2015c':ab,ti OR gp2015:ab,ti OR gp2015c:ab,ti OR 'hd 203':ab,ti OR hd203:ab,ti OR infinitam:ab,ti OR 'lbec 0101':ab,ti OR lbec0101:ab,ti OR lifmior:ab,ti OR nepexto:ab,ti OR opinercept:ab,ti OR 'recombinant tumor necrosis factor receptor fc fusion protein':ab,ti OR 'recombinant tumour necrosis factor receptor fc fusion protein':ab,ti OR reumatocept:ab,ti OR 'sb 4':ab,ti OR sb4:ab,ti OR tnr001:ab,ti OR 'tumor necrosis factor receptor fc fusion protein':ab,ti OR 'tumour necrosis factor receptor fc fusion protein':ab,ti OR tunex:ab,ti OR yisaipu:ab,ti OR 'ylb 113':ab,ti OR ylb113:ab,ti OR 'tnfr-fc fusion protein':ab,ti OR 'fusion protein, tnfr-fc':ab,ti OR 'tnfr fc fusion protein':ab,ti OR 'tnr 001':ab,ti OR 'tnt receptor fusion protein':ab,ti OR 'tntr fc':ab,ti OR 'etanercept szzs':ab,ti OR 'tnf receptor type ii-igg fusion protein':ab,ti OR 'tnf receptor type ii igg fusion protein':ab,ti OR 'recombinant human dimeric tnf receptor type ii-igg fusion protein':ab,ti OR 'recombinant human dimeric tnf receptor type ii igg fusion protein':ab,ti  #24. #22 OR #23  #25. 'itepekimab'/exp  #26. regn3500:ab,ti OR 'regn 3500':ab,ti OR sar440340:ab,ti OR 'sar 440340':ab,ti OR itepekimab*:ab,ti  #27. #25 OR #26  #28. 'astegolimab'/exp OR 'amg 282':ab,ti OR amg282:ab,ti OR mstt1041a:ab,ti OR 'mstt 1041a':ab,ti OR 'ro 7187807':ab,ti OR ro7187807:ab,ti OR rg6149:ab,ti OR 'rg 6149':ab,ti OR astegolimab*:ab,ti  #29. 'astegolimab'/exp  #30. #28 OR #29  #31. 'lebrikizumab'/exp  #32. rg3637:ab,ti OR 'ro 5490255':ab,ti OR 'rg 3637':ab,ti OR 'tnx 650':ab,ti OR milr1444a:ab,ti OR 'milr 1444a':ab,ti OR pro301444:ab,ti OR 'pro 301444':ab,ti OR lebrikizumab*:ab,ti  #33. #31 OR #32  #34. cnto6785:ab,ti  #35. 'amg 282':ab,ti  #36. medi8986:ab,ti  #37. 'tozorakimab'/exp  #38. tozorakimab*:ab,ti OR 'medi 3506':ab,ti OR medi3506:ab,ti  #39. #37 OR #38  #40. 'dupilumab'/exp  #41. dupilumab:ti,ab,kw OR sar231893:ab,ti OR 'sar 231893':ab,ti OR dupixent:ab,ti OR regn668:ab,ti OR 'regn 668':ab,ti  #42. #40 OR #41  #43. #6 OR #9 OR #12 OR #15 OR #18 OR #21 OR #24 OR #27 OR #30 OR #33 OR #34 OR #35 OR #36 OR #39 OR #42  #44. 'crossover procedure':de,ab,ti OR 'double-blind procedure':de,ab,ti OR 'randomized controlled trial':de,ab,ti OR 'single-blind procedure':de,ab,ti OR random*:ab,ti OR factorial*:ab,ti OR crossover*:ab,ti OR ((cross NEXT/1 over*):ab,ti) OR placebo*:ab,ti OR ((doubl* NEAR/1 blind*):ab,ti) OR ((singl* NEAR/1 blind*):ab,ti) OR assign*:ab,ti OR allocat*:ab,ti OR volunteer*:ab,ti  #45. 'formoterol fumarate':ti,ab,kw OR ('3 formylamino 4 hydroxy alpha':ab,ti AND 'n 1 methyl 2 p methoxyphenethylaminomethyl':ab,ti AND benzylalcohol.hemifumarate:ab,ti) OR 'bd 40a':ab,ti OR formoterol:ab,ti OR eformoterol:ab,ti OR oxis:ab,ti OR arformoterol:ab,ti OR foradil:ab,ti  #46. 'formoterol fumarate'/exp  #47. #45 OR #46  #48. 'aclidinium bromide':ti,ab,kw OR (3r:ab,ti AND -3-:ab,ti AND hydroxy:ab,ti AND 'di 2 thienyl':ab,ti AND acetyl:ab,ti AND oxy:ab,ti AND -1-:ab,ti AND '3 phenoxypropyl':ab,ti AND '1 azoniabicyclo':ab,ti AND 2.2.2:ab,ti AND 'octane bromide':ab,ti) OR aclidinium*:ab,ti  #49. 'aclidinium bromide'/exp  #50. #48 OR #49  #51. glycopyrrolate:ti,ab,kw OR 'glycopyrronium bromide':ab,ti OR glycopyrronium:ab,ti OR ('pyrrolidinium, 3-':ab,ti AND cyclopentylhydroxyphenylacetyl:ab,ti AND oxy:ab,ti AND '-1,1-dimethyl-, bromide':ab,ti) OR nva237:ab,ti OR glycopyrronium*:ab,ti  #52. 'glycopyrronium'/exp  #53. #51 OR #52  #54. indacaterol:ti,ab,kw OR indacaterol*:ab,ti OR 'qab 149':ab,ti OR ('pyrrolidinium, 3-':ab,ti AND cyclopentylhydroxyphenylacetyl:ab,ti AND oxy:ab,ti AND '-1,1-dimethyl-, bromide':ab,ti) OR 'arcapta neohaler':ab,ti OR onbrez:ab,ti  #55. 'indacaterol'/exp  #56. #54 OR #55  #57. vilanterol:ti,ab,kw OR vilanterol*:ab,ti OR (4-:ab,ti AND 6-:ab,ti AND 2-:ab,ti AND '2,6 dichlorophenyl':ab,ti AND methyl:ab,ti AND ethyl:ab,ti AND oxy:ab,ti AND hexyl:ab,ti AND amino:ab,ti AND '1 hydroxyethyl':ab,ti AND -2-:ab,ti AND hydroxymethyl:ab,ti AND phenol:ab,ti)  #58. 'vilanterol'/exp  #59. #57 OR #58  #60. gsk573719:ti,ab,kw OR umeclidinium*:ab,ti OR umeclidinium:ab,ti  #61. 'umeclidinium'/exp  #62. #60 OR #61  #63. olodaterol:ti,ab,kw OR olodaterol:ab,ti OR olodaterol*:ab,ti OR ('6 hydroxy 8':ab,ti AND '1 hydroxy 2':ab,ti AND 2-:ab,ti AND '4 methoxyphenyl':ab,ti AND '1,1 dimethylethyl':ab,ti AND amino:ab,ti AND ethyl:ab,ti AND '2h 1,4 benzoxazin 3':ab,ti AND 4h:ab,ti AND -one:ab,ti) OR 'bi 1744':ab,ti OR 'striverdi respimat':ab,ti  #64. 'olodaterol'/exp  #65. #63 OR #64  #66. 'tiotropium bromide':ti,ab,kw OR (7-:ab,ti AND hydroxybis:ab,ti AND '2 thienyl':ab,ti AND acetyl:ab,ti AND oxy:ab,ti AND '9,9 dimethyl 3 oxa 9 azoniatricyclo':ab,ti AND 3.3.1.0:ab,ti AND 2,4:ab,ti AND 'nonane bromide':ab,ti) OR tiotropium:ab,ti OR tiotropium*:ab,ti OR spiriva:ab,ti OR 'ba 679 br':ab,ti  #67. 'tiotropium bromide'/exp  #68. #66 OR #67  #69. budesonide:ti,ab,kw OR budesonide*:ab,ti OR rhinocort:ab,ti OR horacort:ab,ti OR (budesonide,:ab,ti AND r:ab,ti AND -isomer:ab,ti) OR (budesonide,:ab,ti AND s:ab,ti AND -isomer:ab,ti) OR pulmicort:ab,ti OR tarpeyo:ab,ti  #70. 'budesonide'/exp  #71. #69 OR #70  #72. #47 OR #50 OR #53 OR #56 OR #59 OR #62 OR #65 OR #68 OR #71  #73 #43 OR #72  #74. #3 AND #44 AND #73 |
| 1. Search strategies of Cochrane Library |
| #1. MeSH descriptor: [Pulmonary Disease, Chronic Obstructive] explode all trees  #2. (Chronic Obstructive Lung Disease OR Chronic Obstructive Pulmonary Diseases OR COAD OR COPD OR COBD OR Chronic Obstructive Airway Disease OR Chronic Obstructive Pulmonary Disease OR Airflow Obstruction, Chronic OR Airflow Obstructions, Chronic OR Chronic Airflow Obstructions OR Chronic Airflow Obstruction OR Chronic Obstructive Lung Disease):ti,ab,kw (Word variations have been searched)  #3. #1 OR #2  #4. (mepolizumab OR Bosatria OR SB-240563 OR SB240563 OR Nucala OR mepolizumab*):ti,ab,kw (Word variations have been searched)  #5. (reslizumab OR Cinquil OR Cinqair OR SCH-55700 OR SCH55700 OR SCH 55700 OR CEP-38072 OR CEP38072 OR DCP-835 OR DCP835 OR DCP 835 OR reslizumab*):ti,ab,kw (Word variations have been searched)  #6. (benralizumab OR benralizumab* OR MEDI-563 OR MEDI 563 OR Fasenra OR BIW-8405):ti,ab,kw (Word variations have been searched)  #7. (canakinumab OR Ilaris OR ACZ-885 OR ACZ885 OR Canakinumab* OR immunoglobulin G1 OR anti-human interleukin 1beta OR anti-human clone ACZ885 heavy chain V region):ti,ab,kw (Word variations have been searched)  #8. (ABX-Interleukin 8 OR ABX-IL8 MAb):ti,ab,kw (Word variations have been searched)  #9. MeSH descriptor: [Infliximab] explode all trees  #10. (MAb cA2 OR Monoclonal Antibody cA2 OR Antibody cA2, Monoclonal OR cA2, Monoclonal Antibody OR Infliximab-dyyb OR Infliximab dyyb OR Inflectra OR Remicade OR Infliximab-abda OR Infliximab abda OR Renflexis OR Infliximab*):ti,ab,kw (Word variations have been searched)  #11. #9 OR #10  #12. MeSH descriptor: [Etanercept] explode all trees  #13. (TNFR-Fc Fusion Protein OR Fusion Protein, TNFR-Fc OR TNFR Fc Fusion Protein OR TNR-001 OR TNR001 OR TNR 001 OR TNT Receptor Fusion Protein OR TNTR-Fc OR Etanercept-szzs OR TNF Receptor Type II-IgG Fusion Protein OR TNF Receptor Type II IgG Fusion Protein OR Erelzi OR Recombinant Human Dimeric TNF Receptor Type II-IgG Fusion Protein OR Recombinant Human Dimeric TNF Receptor Type II IgG Fusion Protein OR Enbrel):ti,ab,kw (Word variations have been searched)  #14. #12 OR #13  #15. (itepekimab OR REGN-3500 OR REGN3500 OR SAR-440340 OR SAR440340 OR Itepekimab*):ti,ab,kw (Word variations have been searched)  #16. (astegolimab OR MSTT1041A OR MSTT-1041A OR RO-7187807 OR RO7187807 OR RG6149 OR RG-6149 OR Astegolimab*):ti,ab,kw (Word variations have been searched)  #17. (lebrikizumab OR RO-5490255 OR RG-3637 OR TNX-650 OR MILR1444A OR MILR-1444A OR PRO301444 OR PRO-301444 OR Lebrikizumab*):ti,ab,kw (Word variations have been searched)  #18. (CNTO6785):ti,ab,kw (Word variations have been searched)  #19. (AMG 282):ti,ab,kw (Word variations have been searched)  #20. (MEDI8986):ti,ab,kw (Word variations have been searched)  #21. (Tozorakimab or Tozorakimab* or MEDI3506):ti,ab,kw (Word variations have been searched)  #22. (dupilumab):ti,ab,kw OR (SAR231893):ti,ab,kw OR (SAR-231893):ti,ab,kw OR (Dupixent):ti,ab,kw OR (REGN668):ti,ab,kw (Word variations have been searched)  #23. (Formoterol Fumarate):ti,ab,kw OR (Oxis):ti,ab,kw OR (BD 40A):ti,ab,kw OR (Formoterol):ti,ab,kw OR (Eformoterol):ti,ab,kw (Word variations have been searched)  #24. MeSH descriptor: [Formoterol Fumarate] explode all trees  #25. #23 OR #24  #26. (Arformoterol):ti,ab,kw OR Foradil:ti,ab,kw  #27. aclidinium bromide:ti,ab,kw OR aclidinium*:ti,ab,kw  #28. MeSH descriptor: [Tropanes] explode all trees  #29. #26 OR #27 OR #28  #30. Glycopyrronium Bromide:ti,ab,kw OR Glycopyrronium:ti,ab,kw OR NVA237:ti,ab,kw OR glycopyrronium*:ti,ab,kw  #31. MeSH descriptor: [Glycopyrrolate] explode all trees  #32. #30 OR #31  #33. indacaterol*:ti,ab,kw OR QAB-149:ti,ab,kw OR arcapta neohaler:ti,ab,kw OR Onbrez:ti,ab,kw  #34. vilanterol*:ti,ab,kw  #35. umeclidinium*:ti,ab,kw OR umeclidinium:ti,ab,kw OR GSK573719:ti,ab,kw  #36. olodaterol:ti,ab,kw OR Olodaterol*:ti,ab,kw OR Striverdi Respimat:ti,ab,kw OR BI 1744 CL:ti,ab,kw  #37. Tiotropium Bromide:ti,ab,kw OR Tiotropium:ti,ab,kw OR Tiotropium*:ti,ab,kw OR Spiriva:ti,ab,kw OR BA 679 BR:ti,ab,kw  #38. MeSH descriptor: [Tiotropium Bromide] explode all trees  #39. #37 OR #38  #40. budesonide:ti,ab,kw OR budesonide*:ti,ab,kw OR Rhinocort:ti,ab,kw OR Horacort:ti,ab,kw OR Pulmicort:ti,ab,kw OR Tarpeyo:ti,ab,kw  #41. MeSH descriptor: [Budesonide] explode all trees  #42. #40 OR #41  #43 #4 OR #5 OR #6 OR #7 OR #8 OR #11 OR #14 OR #15 OR #16 OR #17 OR #18 OR #19 OR #20 OR #21 OR #22  #44. #25 OR #29 OR #32 OR #33 OR #34 OR #35 OR #36 OR #39 #42  #45. #43 OR #44  #46. #3 AND #45 |

Table S2 Network meta-analysis comparisons for the incidence of TEAEs


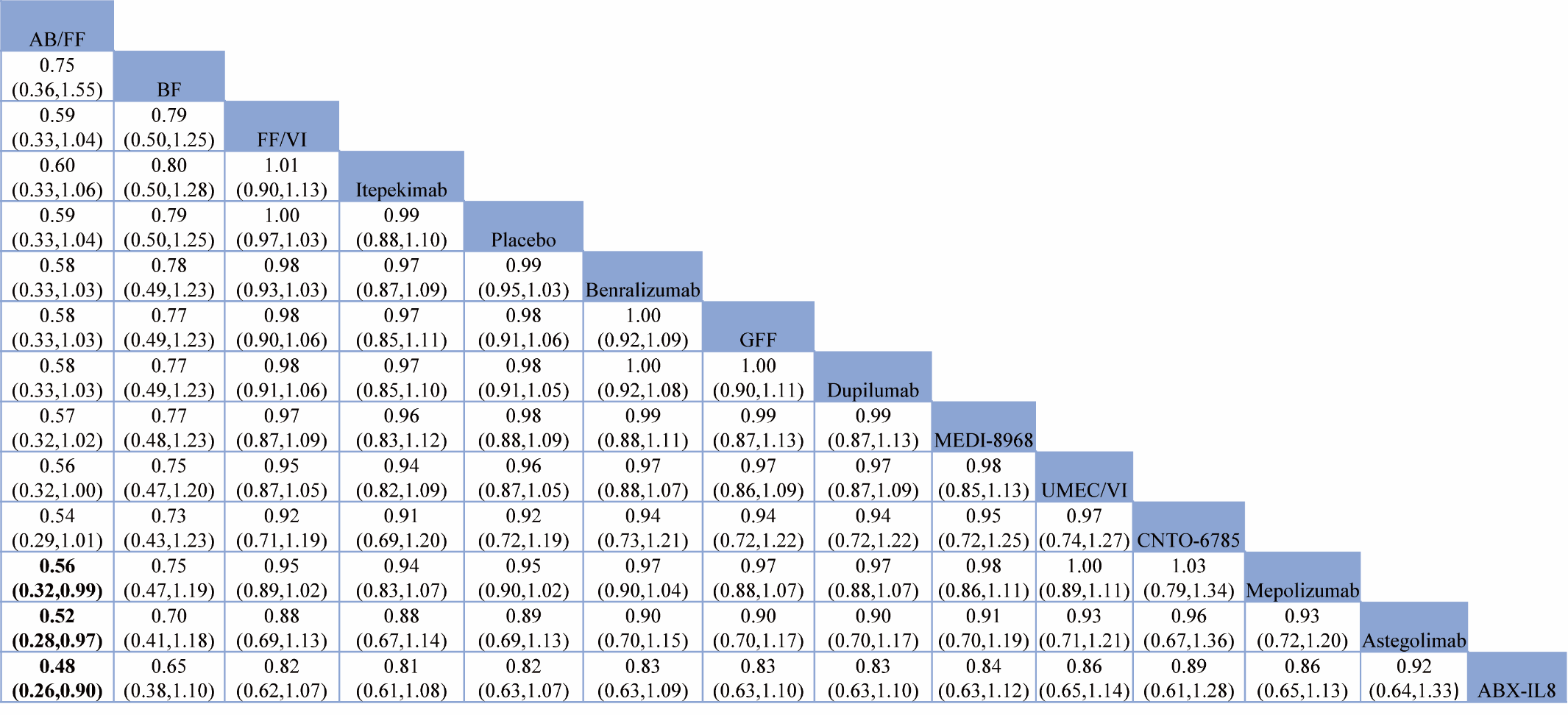


Table S3 Incidence of adverse events

| **Author, Year** | **NCT number** | **Treatment** | **Patient number** | **Patients with ≥1 TEAEs** | | **Patients with TEAEs related to study treatment** | | **Patients with ≥1 serious TEAEs** | | **Patients with serious TEAEs related to study treatment** | | **On-**  **treatment mortality** | |
| --- | --- | --- | --- | --- | --- | --- | --- | --- | --- | --- | --- | --- | --- |
|  |  |  | N | n | % | n | % | n | % | n | % | n | % |
| [Koopman, 2022](https://pubmed.ncbi.nlm.nih.gov/?term=Koopman%20M%5bAuthor%5d) | NCT02424344 | AB/FF 400/12 ug BID | 134 | 16 | 11.9 | NR | NR | NR | NR | NR | NR | NR | NR |
|  |  | Placebo | 133 | 27 | 20.3 | NR | NR | NR | NR | NR | NR | NR | NR |
| [Lipworth, 2018](https://pubmed.ncbi.nlm.nih.gov/?term=Koopman%20M%5bAuthor%5d) | NCT02343458 | GP/FF18/9.6 ug BID | 551 | 306 | 55.5 | 55 | 10 | 53 | 9.6 | 3 | 0.5 | 1 | 0.2 |
|  |  | Placebo | 235 | 131 | 55.7 | 23 | 9.8 | 19 | 8.1 | 2 | 0.9 | 1 | 0.4 |
| Reisner,  2017 | NCT02347072 | GP/FF18/9.6 ug BID | 75 | 19 | 25.3 | 7 | 9.3 | 2 | 2.7 | 3 | 4 | 0 | 0 |
|  |  | Placebo | 72 | 15 | 20.8 | 4 | 5.6 | 2 | 2.8 | 1 | 1 | 0 | 0 |
|  | NCT02347085 | GP/FF18/9.6 ug BID | 40 | 7 | 17.5 | 0 | 0 | 1 | 2.5 | 1 | 2.5 | 0 | 0 |
|  |  | Placebo | 40 | 10 | 25.0 | 1.0 | 2.5 | 1 | 2.5 | 1 | 2.5 | 0 | 0 |
| Martinez, 2017 | NCT01854645 | GP/FF18/9.6 ug BID | 526 | 331 | 62.9 | 65 | 12.4 | 44 | 8.4 | 3 | 0.3 | 4 | 0.8 |
|  |  | Placebo | 219 | 138 | 62.7 | 31 | 14.1 | 16 | 8.1 | 1 | 0.5 | 0 | 0 |
|  | NCT01854658 | GP/FF 8/9.6 ug BID | 510 | 286 | 56.1 | 52 | 10.2 | 36 | 7.1 | 4 | 0.8 | 1 | 0.2 |
|  |  | Placebo | 223 | 117 | 52.5 | 15 | 6.7 | 15 | 6.7 | 0 | NR | 1 | 0.4 |
| Zheng,  2015 | NCT01636713 | UMEC/VI 125/25 ug QD | 193 | 66 | 34.2 | 9 | 5 | 5 | 3 | 0 | 0 | 1 | 0.5 |
|  |  | UMEC/VI 62.5/25 ug QD | 194 | 65 | 33.5 | 7 | 3.6 | 14 | 7.2 | 1 | 0.5 | 1 | 0.5 |
|  |  | placebo | 193 | 76 | 39.3 | 10 | 2.6 | 17 | 8.9 | 1 | 0.5 | 0 | 0 |
| Maltais, 2014 | NCT01323660 | UMEC/VI 125/25 ug QD | 128 | 52 | 40.6 | NR | NR | NR | NR | NR | NR | NR | NR |
|  | NCT01328444 | UMEC/VI 62.5/25 ug QD | 130 | 57 | 43.8 | NR | NR | NR | NR | NR | NR | NR | NR |
|  |  | placebo | 151 | 59 | NR | NR | NR | NR | NR | NR | NR | NR | NR |
| Celli, 2014 | NCT01313637 | UMEC/VI 125/25 ug QD | 403 | 211 | 52.3 | 36 | 8.9 | 17 | 4.2 | 0 | 0 | 2 | 0.5 |
|  |  | placebo | 275 | 134 | 48.7 | 12 | 4.3 | 23 | 8.3 | 0 | 0 | 0 | 0 |
| Donohue, 2013 | NCT01313650 | UMEC/VI 62.5/25 ug QD | 413 | 212 | 50.1 | NR | NR | NR | NR | 21 | 5.1 | 3 | 0.7 |
|  |  | placebo | 280 | 130 | 46.4 | NR | NR | NR | NR | 9 | 3.2 | 1 | 0.3 |
| Tashkin, 2008 | NCT00206154 | BUD/FM 320/9 ug BID | 277 | 21 | 7.6 | 24 | 8.7 | NR | NR | 31 | 11.2 | 3 | 1.1 |
|  |  | BUD/FM 160/9 ug BID | 281 | 20 | 7.1 | 24 | 8.5 | NR | NR | 30 | 10.7 | 4 | 1.4 |
|  |  | placebo | 300 | 28 | 9.3 | 18 | 6 | NR | NR | 25 | 8.3 | 1 | 0.3 |
| [Mahler, 2004](https://journal.chestnet.org/article/S0012-3692(15)31238-1/fulltext) | NR | ABX-IL8 10 mg/mL 3/4w | 59 | 42 | 71.2 | 12 | 20.3 | 3 | 5.1 | NR | NR | NR | NR |
|  |  | placebo | 60 | 35 | 58.3 | 8 | 13.3 | 6 | 10 | NR | NR | NR | NR |
| Brightling, 2014 | NCT01227278 | benralizumab 100 mg Q4w, 8 weeks ago, Q8w 8 weeks later | 51 | 45 | 88.2 | NR | NR | 14 | 27.5 | NR | NR | 2 | 3.9 |
|  |  | placebo | 50 | 41 | 82 | NR | NR | 9 | 18 | NR | NR | 1 | 2 |
| Pavord, 2021 | NCT02105948 | mepolizumab 100mg Q4W | 456 | 281 | 61.6 | NR | NR | 122 | 26.8 | NR | NR | 10 | 2.2 |
|  |  | Placebo | 455 | 274 | 60.2 | NR | NR | 148 | 32.5 | NR | NR | 17 | 3.8 |
|  | NCT02105961 | mepolizumab 300mg Q4W | 225 | 196 | 87.1 | NR | NR | 57 | 25.3 | NR | NR | 8 | 3.5 |
|  |  | Placebo | 226 | 185 | 81.9 | NR | NR | 68 | 30,1 | NR | NR | 9 | 4 |
| Eich,  2017 | NCT01966549 | CNTO 6785 6 mg/kg (>100 kg, 600 mg), 0、2、4、8、12w | 92 | 54 | 58.7 | 9 | 9.8 | 6 | 6.5 | 0 | 0 | 0 | 0 |
|  |  | Placebo | 94 | 51 | 54.3 | 4 | 4.3 | 7 | 7.4 | 0 | 0 | 0 | 0 |
| Calverley, 2017 | [NCT01448850](https://clinicaltrials.gov/ct2/show/NCT01448850) | MEDI8968 600 mg(loading dose), 300 mg Q4W | 160 | 130 | 81.3 | NR | NR | 41 | 25.6 | NR | NR | 6 | 3.8 |
|  |  | Placebo | 164 | 130 | 79.3 | NR | NR | 35 | 21.3 | NR | NR | 3 | 1.8 |
| Dasgupta, 2017 | NCT01463644 | Meperimumab 750mg/month | NR | NR | NR | NR | NR | NR | NR | NR | NR | NR | NR |
|  |  | Placebo | NR | NR | NR | NR | NR | NR | NR | NR | NR | NR | NR |
| Criner  ,2019 | [NCT02138916](http://clinicaltrials.gov/show/NCT02138916)  [NCT02155660](http://clinicaltrials.gov/show/NCT02155660) | Benralizumab 10 mg Q4w 12weeks ago, Q8w later | 561 | 395 | 70.4 | NR | NR | 144 | 25.7 | NR | NR | 17 | 3.0 |
|  |  | Benralizumab 30 mg Q4w 12weeks ago, Q8w later | 1117 | 851 | 76.2 | NR | NR | 328 | 29.4 | NR | NR | 36 | 3.2 |
|  |  | Benralizumab100 mg Q4w 12weeks ago, Q8w later | 1114 | 842 | 75.6 | NR | NR | 304 | 27.3 | NR | NR | 28 | 2.5 |
|  |  | Placebo | 1118 | 827 | 74 | NR | NR | 334 | 29.9 | NR | NR | 32 | 2.9 |
| Bhatt,  2023 | NCT03930732 | dupilumab 300 mg Q2w | 469 | 363 | 77.4 | NR | NR | 64 | 13.6 | NR | NR | 7 | 1.5 |
|  |  | Placebo | 470 | 357 | 76.0 | NR | NR | 73 | 15.5 | NR | NR | 8 | 1.7 |
| Vestbo,  2016 | NCT01313676 | FF/VI 100/25 ug QD | 4140 | 2780 | 67.1 | NR | NR | 961 | 23.2 | NR | NR | 182 | 4.3 |
|  |  | Placebo | 4131 | 2782 | 67.3 | NR | NR | 918 | 22.2 | NR | NR | 192 | 4.6 |
| Rabe,  2021 | NCT03546907 | Itepekimab 300 mg Q2w | 172 | 135 | 78.5 | NR | NR | 29 | 16.9 | NR | NR | 3 | 1.7 |
|  |  | Placebo | 171 | 136 | 79.5 | NR | NR | 36 | 21.1 | NR | NR | 2 | 1.2 |
| Yousuf,  2022 | NCT03615040 | astegolimab 490 mg Q4W | 42 | 34 | 80.1 | NR | NR | 12 | 28.6 | NR | NR | 0 | 0 |
|  |  | Placebo | 39 | 28 | 71.8 | NR | NR | 16 | 41 | NR | NR | 2 | 5.1 |


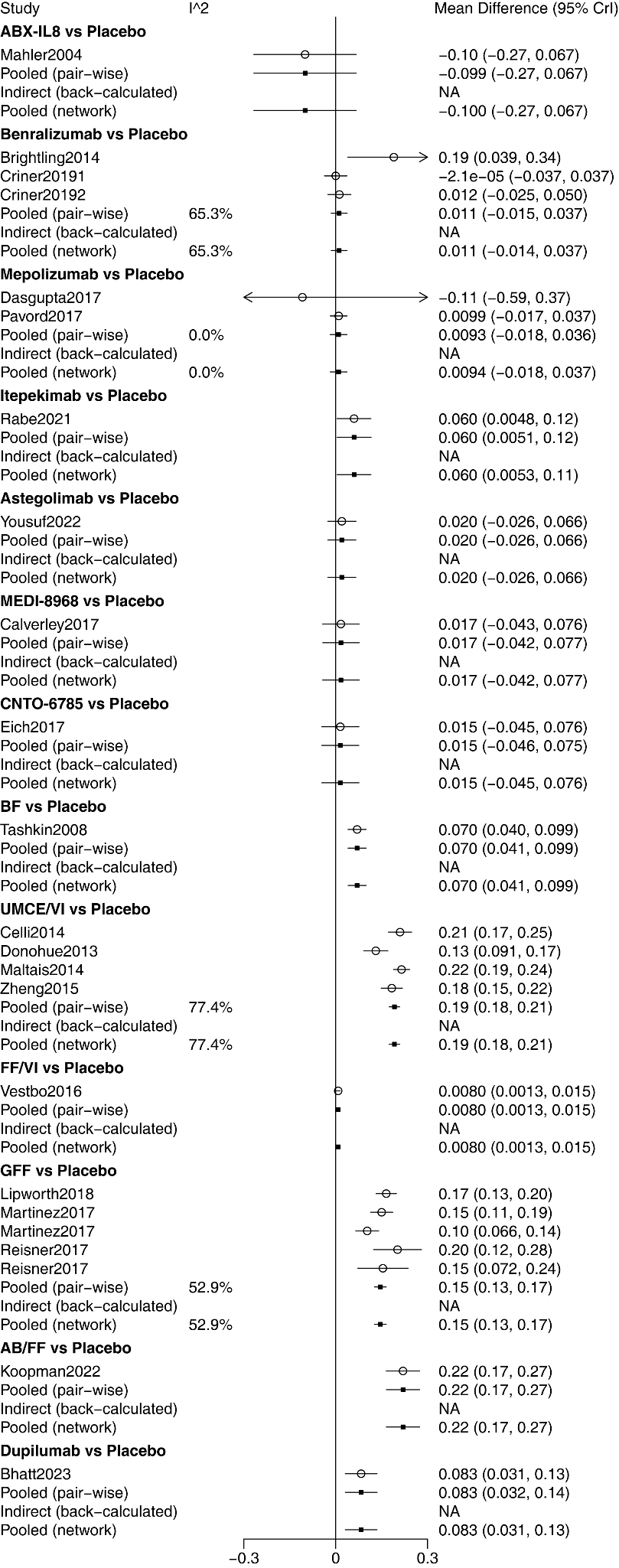


Figure S1 Forest plot for results of FEV1 improvement


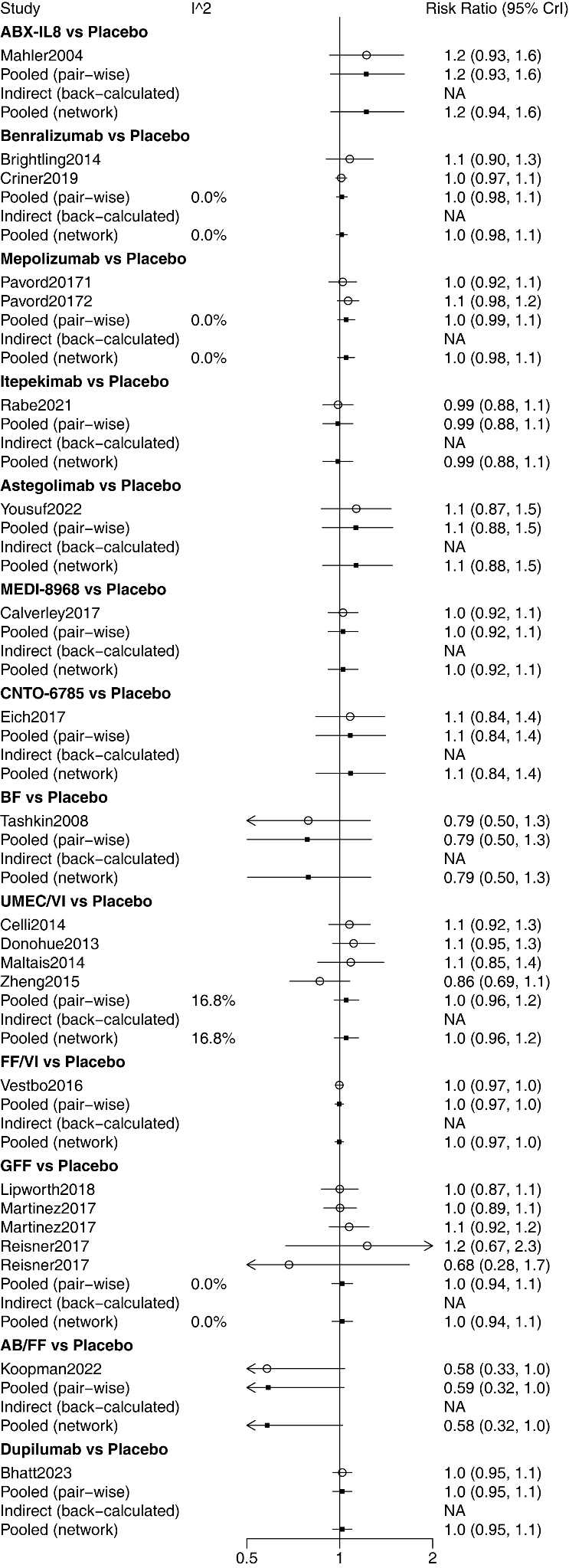


Figure S2 Forest plot for the incidence of TEAEs
